# Efficacy and *in vitro* pharmacological assessment of novel *N*-hydroxypyridinediones as hepatitis B virus ribonuclease H inhibitors

**DOI:** 10.1128/aac.01455-24

**Published:** 2024-11-27

**Authors:** Molly E. Woodson, Holly F. Walden, M. Abdul Mottaleb, Maria Makri, Georgia-Myrto Prifti, Dimitrios Moianos, Vasiliki Pardali, Grigoris Zoidis, John E. Tavis

**Affiliations:** 1 Department of Molecular Microbiology and Immunology, St. Louis University School of Medicine 12274 , St. Louis, Missouri, USA; 2 Institute for Drug and Biotherapeutic Innovation, St. Louis University 7547 , St. Louis, Missouri, USA; 3 Division of Pharmaceutical Chemistry, Department of Pharmacy, School of Health Sciences, National and Kapodistrian University of Athens, Panepistimiopolis Zografou, Athens, Greece; IrsiCaixa Institut de Recerca de la Sida, Barcelona, Spain

**Keywords:** HBV, *N*-hydroxypyridinedione, pharmacology, drug discovery

## Abstract

We previously reported *N*-hydroxypyridinedione (HPD) compounds with mid-nanomolar efficacy and selectivity indexes around 300 against hepatitis B virus (HBV) replication. However, they lack pharmacological evaluation. Here, we report *in vitro* anti-HBV efficacy, cytotoxicity, and pharmacological characterization of 29 novel HPDs within seven subgroups. The best two compounds had EC_50_s of 61 and 190 nM and selectivity indexes of 526 and 1,071. Compounds with one halogen on the major R group were most effective and compounds with large ether R groups were most cytotoxic. Compounds were not cytotoxic in primary human hepatocytes. All compounds were freely soluble in pHs reflecting plasma (7.4) and the gastrointestinal tract (5 and 6.5). Almost all highly soluble compounds were passively permeable at pH 5.0 and 7.4. Only 2 of 11 compounds tested were likely to be effluxed by p-glycoprotein. The most potent HPDs inhibited HBV replication over human ribonuclease H1 activity by 10-fold. Four of 19 compounds inhibited CYP2D6 >50%, but their CYP2D6 IC_50_s were >8× higher than their anti-HBV EC_50_. No compound substantially inhibited CYP3A4. Thirteen of 15 compounds had human microsomal half-lives >30 min with medium to low rates of intrinsic clearance. Eleven of 12 compounds bound plasma proteins by ≥80%; however, effects against HBV replication for only one would likely be physiologically relevant. These results identify two lead candidate HPDs with pharmacological characteristics resembling commercially available drugs that are suitable for *in vivo* pharmacological and efficacy studies.

## INTRODUCTION

Hepatitis B virus (HBV) chronically infects up to 300 million people worldwide, resulting in greater than 1 million deaths annually due to liver complications such as cirrhosis and hepatocellular carcinoma (1). The United States Food and Drug Administration has approved eight drugs for the treatment of chronic hepatitis B including six nucleos(t)ide analogs (NA) and two interferon-α formulations (IFN-α) (2). NAs and IFN-α, either as monotherapy or in combination, can suppress viremia 5–6 log10 in 70–90% of patients, resulting in improved patient outcomes; however, they are not curative, IFN-α therapy often causes serious side effects and treatment with NAs is lifelong (3, 4). Therefore, achieving a functional cure will require the identification and co-administration of novel anti-HBV therapies with new targets (5, 6).

HBV is a hepatotropic, partially double-stranded DNA virus that replicates by reverse transcription. Upon entering hepatocytes via the sodium taurocholate co-transporting polypeptide (NTCP) receptor, the viral relaxed circular DNA (rcDNA) genome is trafficked to the nucleus, where it is repaired to become the episomal covalently closed circular DNA (cccDNA), which serves as the template for all viral transcription (7, 8). Reverse transcription is catalyzed by the viral polymerase (P) protein and is initiated when P binds to the pregenomic RNA (pgRNA) template (9). P has two catalytic domains, the reverse transcriptase (RT) and the ribonuclease H (RNase H) domain (10). The RT domain synthesizes the minus polarity DNA strand from pgRNA, which is concurrently degraded by the RNase H domain, thereby permitting synthesis of the plus polarity DNA strand by the RT. Following reverse transcription, the viral relaxed circular DNA (rcDNA) genome in nucleocapsids is either trafficked to the nucleus to replenish the cccDNA pool or is noncytolytically secreted from the cell as a mature virion (11). NAs targeting the RT active site, tenofovir disoproxil fumarate, tenofovir alafenamide, and entecavir, are recommended as first-line treatment due to their high clinical efficacy with few side effects (12). However, they do not directly impact the cccDNA and only very slowly reduce its levels over years of treatment (13–16), and as such, treatment cessation usually results in viremia recurrence (17, 18).

We have demonstrated that the HBV RNase H domain is an attractive drug target because inhibiting it renders the minus polarity DNA strand useless as a template for the plus polarity strand. Consequently, plus polarity DNA strand synthesis is blocked, resulting in an accumulation of RNA:DNA heteroduplexes (19, 20). HBV RNase H inhibitors also inhibit cccDNA formation and synergistically reduce HBV replication upon co-treatment with NAs (21–23).

The *N*-hydroxypyridinedione (HPD) pharmacophore is defined by a six-membered ring with an oxygen trident, which coordinates the Mg^2+^ ions in the HBV RNase H active site that are essential for its activity (24). Previous work has shown that HPD compounds selectively inhibit HBV replication by preferentially suppressing plus polarity DNA strand synthesis at submicromolar levels (best effective concentration 50% [EC_50_ ] ~300 nM) with minimal cytotoxicity (selectivity index [cytotoxicity/efficacy] >300), but they lack the extensive pharmacological characterization that is needed in preclinical studies (20, 22, 25). Therefore, in support of an ongoing medicinal chemistry campaign to identify lead candidate HPDs, we report on the anti-HBV efficacy and *in vitro* pharmacological evaluation of 29 novel HPDs.

## MATERIALS AND METHODS

### Compound acquisition and synthesis

Compounds were synthesized by Grigoris Zoidis’ group and were determined to be >95% pure by ^1^H-NMR, ^13^C NMR spectra, and elemental analyses (C, H, and N) (see the supplemental material). Upon receipt, compounds were dissolved in 100% dimethyl sulfoxide (DMSO) to 10 mM and stored in small aliquots in opaque tubes at −25°C.

### Cell culture

HepDES19 cells are a hepG2 derivative that carry a tetracycline-repressible HBV genomic expression cassette (26) and were a gift from Dr. Haitao Guo at the University of Pittsburgh. HepG2 cells were obtained from ATCC and MDCK-MDR1 cells were a gift from Dr. David Griggs at Saint Louis University. HepDES19 cells were maintained in the presence of 10 µg/mL tetracycline. All cell lines were incubated on collagen-coated plates (Corning) at 37°C with saturating humidity in Dulbecco’s modified Eagle’s medium (DMEM)-F12 (Cytiva Life Sciences) supplemented with 10% fetal bovine serum (FBS), penicillin (100 IU/mL), and streptomycin (10 µg/mL). Cryopreserved, plateable, primary human hepatocytes (PHHs) were obtained from Yecuris Corporation and stored in liquid nitrogen until use.

### Cytotoxicity

#### HepDES19

HepDES19 cells were plated on 96-well tissue culture-treated plates (Greiner) at 1.0 × 10^4^ cells per well and incubated for 48 h. Cells were treated with titrated concentrations of compound or 1% DMSO vehicle for 72 h, and cellular viability was measured by 3-(4,5-dimethylthiazol-2-yl)−5-(3-carboxymethoxyphenyl)−2-(4-sulfophenyl)−2H-tetrazolium (MTS) assays, as described previously (20). The 50% cytotoxic concentration (CC_50_) values were determined with GraphPad Prism using the four-parameter log[inhibitor] versus response algorithm with the bottom set to zero. Three or more replicate assays were done on different days.

#### Primary human hepatocytes

PHH cytotoxicity was determined by MTS assays following 72 h compound exposure, as described in Woodson et al. (27). Three or more replicate assays were done on different days.

### Anti-HBV efficacy

HBV replication inhibition was measured in hepDES19 cells following 72 h compound exposure without compound replenishment. EC_50_ values were determined by HBV plus-polarity strand suppression using a strand preferential qPCR assay, as described previously (28). Three or more replicate values were obtained on different days.

### Solubility limit

Compound solubility limits were determined in DMEM-F12 without phenol red (Gibco) supplemented with 10% FBS (pH 7.4) to mimic tissue culture experiments and at pH 5 and 6.5 (Biorelevant) to simulate the fed and fasted intestinal states, respectively. Solubility limits were determined by a turbidimetric assay, as described (27), with the solubility limit being defined as the compound concentration where light scattering began to rise. Two or more replicate assays were performed for each compound at each pH on different days.

### Parallel artificial membrane permeability assay

Apparent passive permeability (P_app_ [cm/s]) through a 1% wt/vol lecithin/dodecane membrane was assessed using a 96-well, donor/acceptor cassette (Sigma Aldrich), as previously described (27) in buffers that mimic plasma (pH 7.4) and the fed intestinal state (pH 5) with incubation for 2 h at room temperature with shaking. Compound P_app_ values were determined by normalizing the experimental compound absorbance to the compounds’ absorbances at equilibrium, membrane porosity, buffer volume, and time (s). Two or more replicate assays were obtained on separate days for each pH.

### MDCK-MDR1 permeability assays

MDCK-MDR1 cells were seeded on 96-well, high protein binding immobilon-P membrane plates (Millipore Sigma) at 2.4 × 10^4^ cells per well for 4 days. Media were then decanted, and the cells were equilibrated with Hanks balanced salt solution (HBSS) supplemented with 10 mM CaCl_2_ and 1 mM MgCl_2_ for 1 h. Compound (10 µM) was then added to the apical side to assess active transport and to the basolateral side to assess active efflux. The cells were incubated with compound for 2 h with shaking at 37°C. Samples (50 µL) were retrieved from the recipient wells and mixed with 150 µL acetonitrile (ACN) and internal standard (1:1 of 200 ng/mL chloridazon:enalapril). Samples were pelleted at 4,000 × *g* and the supernatant was analyzed by liquid-chromatography/tandem mass spectrometry (LCMS/MS) analyses. The analyte peak:internal standard area under the curve (AUC) ratios were calculated, and apparent permeability (P_app_ [cm/s]) was determined for active transport (apical to basolateral wells [A – B]) and efflux (basolateral to apical wells [B – A]) by P_app_ = *Q*_AB,BA_/(*C*_0_ × *s* × *t*), where *Q* is the amount of compound in the acceptor compartment, *C*_0_ is the amount of compound in the donor compartment, *s* is the surface area of the membrane and *t* is the time in seconds (29). Active efflux was determined by P_app_ B/A/P_app_ A/B.

Tight junction formation was assessed by lucifer yellow permeation immediately following sample retrieval. Briefly, lucifer yellow (10 µM) was added to the apical wells and HBSS was added to the basolateral wells for 1 h with shaking at 37°C, after which sample was collected from the basolateral side and read on a Biotek Synergy HTX multi-mode plate reader with 485/20 excitation and 520/20 emission filters defined by the Center Wavelength/band-pass values. The percent lucifer yellow fluorescence was determined by subtracting the HBSS relative fluorescent units (RFU) from the sample RFU and normalizing to the lucifer yellow positive control RFU. Lucifer yellow permeation was determined to be acceptable if ≤3%.

### Human RNase H1 inhibition

Recombinant human ribonuclease H1 (huRNase H1) was expressed in *Escherichia coli* and purified by nickel affinity chromatography, as described previously (30). Reactions were performed on black 384-well plates (Corning) with 12 nM RNA:DNA heteroduplex substrate, 5U RNaseOUT (Thermo Fisher), 1.83 nM recombinant huRNase H1, buffer (500 mM Tris-HCl [pH 7.5], 1 M NaCl, and 20 mM TCEP [pH 7.5]), and titrated concentrations of experimental compounds (100 µM to 5 nM in 5% DMSO) or 5% DMSO. The heteroduplex substrate was prepared, as described by Ponzar et al. (30). Reactions were initiated by the addition of MgCl_2_ (5 mM final) and were performed on a Biotek Synergy HTX multi-mode plate reader at 28°C. Cleavage of the fluorophore-conjugated RNA was detected every 60 s with 485/20 nm and 520/20 nm excitation/emission filters defined by the Center Wavelength/band-pass values.

Maximum rates of fluorescence increase were calculated using the Biotek Gen5 v3.15 software and percentage activity was determined for each compound concentration by subtracting rates from no enzyme reactions and normalizing to DMSO reactions. Dose-response curves were generated in GraphPad Prism and 50% inhibitory concentrations (IC_50_s) were determined using the four-parameter log[inhibitor] versus response algorithm. Reactions were performed at least three times on separate days.

### Cytochrome P450 inhibition

Compound inhibition of Cytochrome P (CYP) 3A4, 2C9, and 2D6 was determined using Vivid CYP450 screening kits (Thermo Fisher) according to the manufacturer’s instructions, as described previously (27). The kits employed were: Vivid 3A4 DBOMF Green, 2C9 BOMF Green, and 2D6 EOMCC Blue. Reactions were initiated by addition of the fluorophore-conjugated substrates with Vivid NADP^+^ and were conducted at 37°C using either a Biotek Synergy HTX multi-mode plate reader with 485/20 nm excitation and 520/20 nm emission filters (green-fluorescent substrate), or a Tecan Spark plate reader with monochromator settings at 415/20 nm excitation and 460/20 nm emission (blue-fluorescent substrate) defined by the Center Wavelength/band-pass values. Substrate cleavage was detected every 60 s.

Compounds were initially screened against each enzyme at 10 µM and maximum rates of fluorescence in RFU were calculated using the Biotek Gen5 v3.11 software. Percent inhibition of experimental compound reactions was normalized to the rate to DMSO vehicle reactions. Positive controls used were: CYP3A4—ketoconazole (Sigma Aldrich), CYP2C9—sulfaphenazole (Cayman Chemical), and CYP2D6—quinidine (Fisher Scientific). Dose-response curves were generated for compounds that inhibited an enzyme by ≥50% using titrated concentrations of compound (50 µM to 3 nM) or 1% DMSO. IC_50_ values were determined using the four-parameter log[inhibitor] versus response algorithm in GraphPad Prism. Qualitative screens and dose-response curves were each performed in triplicate on different days.

### Microsome stability

Human liver microsomes (gender pooled, 50 individuals) were purchased from BioIVT and stored at −80°C until use. The reactions were prepared in 96-well deep-well plates (USA Scientific) and contained 1 µM test compound, human liver microsomes at 0.657 mg/mL of microsomal protein, and potassium phosphate buffer (3.2 mM MgCl_2_, 20.2 mM KH_2_PO_4_, and 120 mM K_2_HPO_4_) in a final volume of 500 µL. Reactions were initiated by addition of 1.2 mM NADPH. Samples (50 µL) were withdrawn at *t* = 0, 5, 10, 20, and 30 min, and were added to 150 µL of ice-cold acetonitrile solution, as described above. Negative control samples without NADPH were retrieved at *t* = 0 and 30 min. Microsomal proteins were pelleted at 4,000 × *g* and the supernatant was analyzed via LCMS/MS. The AUC experimental sample analyte peak: internal standard analyte peak ratios were calculated, normalized to the *t* = 0 min AUC ratio, and converted to natural log (ln) percent remaining. The ln percent remaining values were plotted against time, the negative slope was taken (*k*), and compound half-life (*t*_*1*/2_) was determined using *ln2*/*k*. Intrinsic clearance (Cl_int_ [µL/min/mg]) was calculated by (*V* × *ln2*)/*t*_1/2_, where *V* is the incubation volume (µL) divided by the protein concentration in the reaction (mg) (31). Controls were warfarin (low clearance rate) and ritonavir (high clearance rate).

### Plasma protein binding

Plasma protein binding was performed using the Pierce rapid equilibrium dialysis device (Thermo Fisher) per the manufacturer’s instructions, as described (27). Briefly, the sample wells contained DMEM-F12 supplemented with 10% FBS spiked with 10 µM compound, and the buffer wells contained phosphate-buffered saline (PBS). The plate was incubated for 4 h at 37°C with shaking, after which 50 µL was retrieved from the buffer and sample wells and added to ice-cold acetonitrile and the internal standard described above. Samples were matrix-matched with PBS supplemented with 10% FBS or PBS alone, respectively, and centrifuged at 4,000 × *g* for 5 min to pellet plasma proteins. The supernatants were analyzed by LCMS/MS and percent compound bound was determined, as described previously (27). Controls were warfarin (high percentage binding) and lamivudine (low percentage binding).

### Effect of plasma proteins on compound potency

HBV plus-polarity DNA suppression was assessed in hepDES19 cells following compound treatment for 72 h. Cells incubated with compound (10 µM) in either normal tissue culture media conditions containing 10% FBS, or in media supplemented with bovine serum albumin (BSA) to 40 mg/mL. Percent inhibition was determined, as described previously (27, 28). The presence of increased BSA levels was determined to affect compound potency if there was a statistically significant decrease in percent HBV plus polarity DNA suppression when compared to cells treated with compound in normal media conditions. Three or more replicate assays were performed on separate days.

### CYP3A4 induction

HepG2 cells were seeded on 12-well, tissue-culture-treated plates (Sarstedt) at 2.5 × 10^5^ cells/well for 24 h. Cells were treated with 10 µM compound or 1% DMSO for 48 h with replenishment at 24 h. Cells were rinsed with PBS and lysed at 0, 24, and 48 h using TRI Reagent (Sigma Aldrich). Cellular RNA was isolated using chloroform:isoamyl alcohol, as described (32), precipitated overnight at −20°C with 100% ethanol and 50 mM NaCl, and then washed with 75% ethanol. Cellular DNA was degraded using DNase I (Invitrogen), according to the manufacturer’s instructions. An additional phenol-chloroform extraction was then performed, followed by an overnight precipitation with 100% ethanol and 50 mM NaCl at −20°C and a wash with 75% ethanol. Cellular RNA concentration was determined at 260 nm using a Thermo Fisher NanoDrop One, and purity was determined by the *A*260/280 and *A*260/230 ratios; a ratio of >1.6 was considered acceptable.

Complementary DNA (cDNA) synthesis employed the ProtoScript II First Strand cDNA Synthesis Kit (New England Biolabs) according to the manufacturer’s instructions with an additional incubation step at 25°C for 5 min. Reverse transcriptase-qPCR (RT-qPCR) was performed using a Quantstudio 5 (Thermo Fisher) in a final reaction volume of 20 µL with 4 µL cDNA template, 2× KAPA probe force master mix (Roche), 300 nM probe, and 100 nM forward and reverse primer. CYP3A4 expression was determined by normalizing the experimental sample cycle threshold values to DMSO-treated wells. GAPDH was used as a housekeeping gene. Primer and probe sequences for GAPDH and CYP3A4 are in Table 1.

**TABLE 1:** Primer and probe sequences for CYP3A4 induction

Target		Sequence and location^*a*^
CYP3A4	Forward	CTCTCGGTTTAGGTGAGGTAATG (2590–2613)
	Reverse	GGAGGATCTCCCTCTCTATGTT (2688–2710)
	Probe	5′6-FAM/TGTTCTGTGCATCTCTCAGTGTGGG/3′IABkFQ (2663–2688)
GAPDH	Forward	GTGGTCTCCTCTGACTTCAAC (8409–8439)
	Reverse	CCTGTTGCTGTAGCCAAATTC (8621–8641)
	Probe	5′6-FAM/TTGCCCTCAACGACCACTTTGTCA/3′IABkFQ (8470–8494)

^*a*^ Base number in full gene nucleotide sequence.

### Liquid chromatography-mass spectrometry/mass spectrometry

A benchtop binary Shimadzu LC (model: LC 20 AD) equipped with HTC PAL autosampler interfaced with a Sciex mass spectrometer (MS) (model: API 4000) was used. The LCMS/MS parameters for all experimental compounds, controls, and internal standards were optimized via direct infusion of the standard solution. All experimental compounds and internal standards, except for warfarin, ritonavir, and **1713**, were separated by liquid chromatography and detected by mass spectrometry, as described (27). **1713** was separated using a gradient of 0.1% formic acid in water (A) and ACN (B), beginning with 20% B at 0.1 min, 80% B at 0.2 min, 98% B at 1.5–2.50 min, 20% B at 2.51 min and held the composition for 1.0 min to equilibrate the system. Warfarin and ritonavir were separated using 5% B at 0.02 min, 95% B at 1.0–2.0 min, 5% B at 2.50 min and held the composition for 0.6 min for system equilibration. Data acquisition for ritonavir, warfarin, and **1713** was made using the positive mode. All analyte and internal standard peaks were analyzed using the Analyst software (v 1.6.3).

### Statistics

The significance between HBV plus-polarity DNA suppression values of compounds in media with the presence or absence of additional BSA was determined by multiple unpaired Mann-Whitney *t* tests on GraphPad Prism v.9.1.2. All data are reported as means ± 1 standard deviation.

## RESULTS

### Compound selection

**Fig 1:**
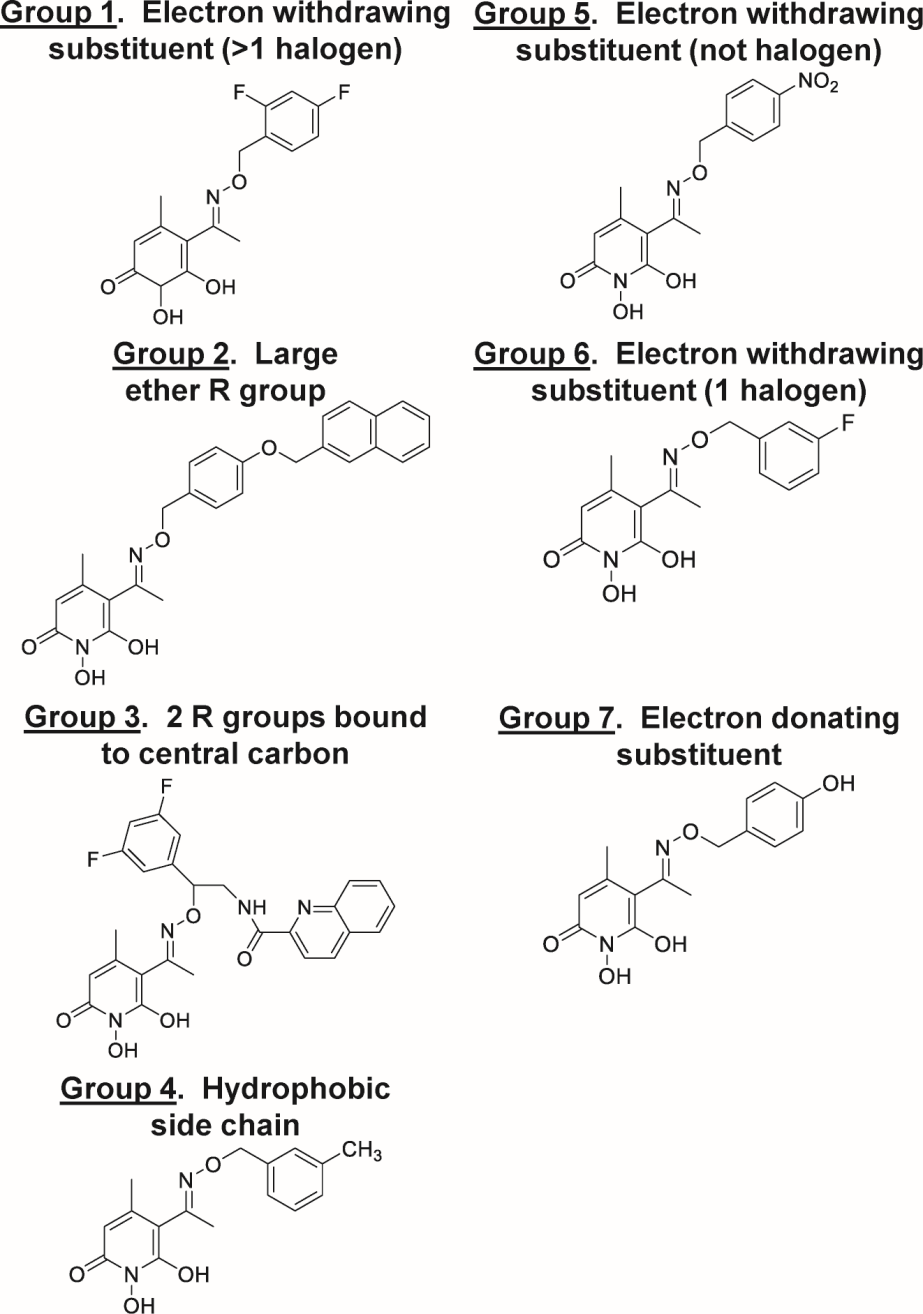
Representative structures of compounds within the seven HPD subgroups.

We previously demonstrated that the oxime *N*-hydroxypyridinediones (HPDs) can inhibit HBV plus polarity DNA strand synthesis at low to submicromolar levels with minimal cytotoxicity (20, 22, 24). Therefore, to guide the medicinal chemistry campaign of these promising anti-HBV compounds, we chose 29 oxime HPD compounds within seven subclasses for in-depth *in vitro* pharmacological analysis. These subclasses are defined by the variable R group off of the oxime bond and are described in Table 2. Representative compound structures are in Fig. 1 and all structures are in Fig. S1.

**TABLE 2:** HPD subclass grouping^*a*^

Group number	Subclass description
1	Electron-withdrawing substituents (>1 halogen)
2	Large ether R group
3	2 R groups bound to central carbon
4	Hydrophobic side chain
5	Electron-withdrawing substituent (not halogen)
6	Electron-withdrawing substituent (one halogen)
7	Electron-donating substituent

^*a*^ Compound subclasses were determined by the similarities in R group.

### HepDES19 efficacy and cytotoxicity

All 29 HPDs were screened for their efficacy in hepDES19 cells, a hepG2 derivative that contains a tetracycline-repressible HBV genome expression cassette in which viral surface glycoprotein expression is ablated (26). Half-maximal effective concentration values (EC_50_) were determined using an established strand-specific qPCR assay following 3 days of compound exposure because RNase H inhibitors preferentially suppress accumulation of the viral plus-polarity DNA strand (28) (Table 3). Compounds within Group 6 (one halogen) were the most potent, having an average EC_50_ of 0.81 µM, and compounds within Group 1 (>one halogen) were the second most potent, with an average EC_50_ = 1.06 µM, while Group 2 (large ether) were the least potent with an EC_50_ average of 5.13 µM. We also identified **1618** in Group 6 (one halogen) as the most potent HBV RNase H inhibitor to date with an EC_50_ of 61 nM.

**TABLE 3:** HPD efficacy and cytotoxicity^*a*^

Compound	HepDES19 EC_50_^*b*^ (µM)	HepDES19 CC_50_^*b*^ (µM)	HepDES19 SI (CC_50_/EC_50_)	PHH CC_50_^*b*^ (µM)
Group 1: Electron-withdrawing substituents (>1 halogen)				
1235	1.87 ± 0.351	49.2 ± 19.2	26.3	−^*c*^
1464	0.188 ± 0.063	63.6 ± 27.0	281	100 ± 0
1617	0.350 ± 0.193	72.9 ± 37.1	157	100 ± 0
1810	1.85 ± 0.758	100 ± 0	54.0	−
Group 2: Large ether R group				
1738	7.49 ± 1.73	51.4 ± 19.2	6.53	−
1808	6.00 ± 0.170	38.2 ± 10.2	6.24	−
1899	1.92 ± 0.700	68.6 ± 24.3	40.7	100 ± 0
Group 3: 2 R groups bound to central carbon				
1680	2.24 ± 0.228	47.3 ± 23.7	21.0	−
1908	3.46 ± 0.935	99.9 ± 0.23	28.9	−
1910	3.33 ± 1.79	100 ± 0	30.0	100 ± 0
Group 4: Hydrophobic side chain				
1462	0.295 ± 0.122	33.1 ± 5.24	112	100 ± 0
1620	0.19 ± 0.087	100 ± 0	526	100 ± 0
1681	3.97 ± 1.19	100 ± 0	25.2	−
1714	2.48 ± 0.777	53.6 ± 36.3	15.3	−
1811	1.40 ± 1.19	64.3 ± 25.2	71.3	−
Group 5: Electron−withdrawing substituent—not a halogen				
1619	1.14 ± 0.469	100 ± 0	87.9	100 ± 0
1622	1.40 ± 0.800	100 ± 0	71.6	84.5 ± 15.5
1737	7.74 ± 1.75	91.9 ± 13.7	11.9	−
1895	1.09 ± 0.298	99.6 ± 0.75	91.7	−
Group 6: Electron-withdrawing substituent—one halogen				
1463	0.240 ± 0.091	58.2 ± 29.1	186	100 ± 0
1466	0.359 ± 0.240	46.6 ± 13.6	130	−
1618	0.061 ± 0.010	73.9 ± 28.6	1,071	93.8 ± 10.7
1621	0.52 ± 0.330	78.5 ± 32.4	192	100 ± 0
1717	1.13 ± 0.186	76.7 ± 30.3	52.7	−
1718	2.00 ± 1.63	80.8 ± 27.5	34.1	−
1719	1.35 ± 1.03	56.9 ± 31.1	34.5	−
Group 7: Electron-donating substituent				
1669	1.13 ± 0.955	91.0 ± 15.6	80.6	100 ± 0
1670	1.92 ± 1.42	90.0 ± 17.3	47.0	−
1713	5.15 ± 1.07	91.1 ± 16.5	17.1	−

^*a*^ Data are represented as the mean ± standard deviation from three independent experiments.

^*b*^ The highest concentration tested in EC_50_ and CC_50_ experiments was 100 µM.

^*c*^ −, not tested.

Cytotoxic concentration 50% (CC_50_) values were determined by MTS assays with the same 29 HPDs in hepDES19 cells after incubation with compound for 72 h (Table 3). Compounds within Group 5 (electron-withdrawing substituent, not halogen) were the least cytotoxic with an average CC_50_ 97.5 µM, and compounds within Group 2 (large ether R group) were the most cytotoxic, having an average CC_50_ of 52.7 µM. These data indicate that compound efficacy and cytotoxicity are both subclass- and compound-specific.

### Primary human hepatocyte cytotoxicity

We screened 12 structurally diverse compounds within the seven subclasses (**1464** and **1617** [Group 1: >1 halogen], **1899** [Group 2: large ether], **1910** [Group 3: 2 R groups], **1462** and **1620** [Group 4: hydrophobic], **1619** and **1622** [Group 5: not halogen], **1463**, **1618**, **1621** [Group 6: one halogen], and **1669** [Group 7: donating]) for their cytotoxicity in primary human hepatocytes following 72 h compound exposure (Table 3). CC_50_ values were determined by MTS assays. These compounds were chosen because they were either the most potent compounds within their subclasses or were minimally cytotoxic in hepDES19 cells. Ten of 12 compounds were not cytotoxic in PHHs (CC_50_ >100 µM), and two compounds (**1622** [Group 5] and **1618** [Group 6]) were minimally toxic (CC_50_s = 84.5 and 93.8 µM, respectively). Therefore, compounds are generally much less cytotoxic in PHHs than in hepDES19s.

### Solubility limit

**Fig 2:**
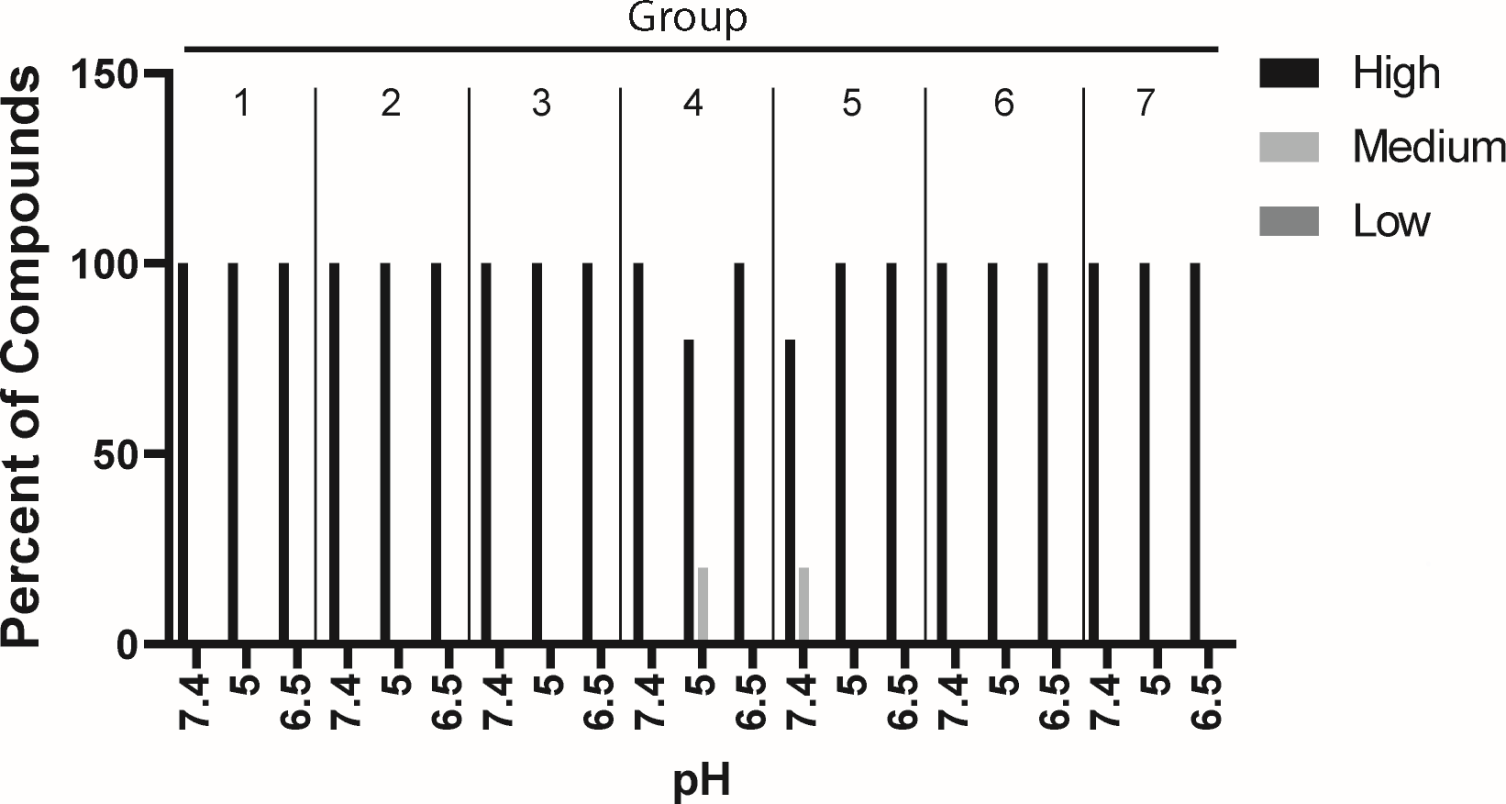
Effect of pH on HPD solubility limits. Compounds were serially diluted in buffers that reflect the GI tract pH: 7.4 (plasma), 5.0 (fed simulated intestinal state), and 6.5 (fasted simulated intestinal state). Solubility limits were determined to be high >100 µM, partial 100–1 µM, and insoluble <1 µM. *N* > 2.

The gastrointestinal (GI) tract has a pH range of 1.6–7.4 depending on position within the tract (33, 34). Therefore, all 29 compounds were evaluated for their likelihood to precipitate in biological fluids by testing their solubility limits at physiologically relevant pHs: fed simulated intestinal fluid (FessIF: 5.0) and fasted simulated intestinal fluid (FassIF: 6.5), which were selected because the majority of drug absorption occurs in the small intestine, and DMEM-F12 without phenol red supplemented with 10% FBS (7.4) to reflect tissue culture conditions, using a turbidimetric assay (Fig. 2; individual values in Table S1). Solubility limits were classified into three categories: Highly soluble (≥100 µM), partially soluble (100–1 µM), and insoluble (<1 µM), according to the industry standard categories (35, 36). Compounds **1811** (Group 4) and **1717** (Group 6) were partially soluble with limits of 50 µM at pH 5.0 and 6.5, respectively; however, 50 µM is in the middle of this range, and thus these compounds are still very soluble. All 29 compounds screened at pH 7.4 had high solubility limits. These data indicate that solubility limits were compound-specific instead of R group-subclass specific, with HPDs being more soluble at a pH that mimicked plasma (7.4).

### Parallel artificial membrane permeability

**Fig 3:**
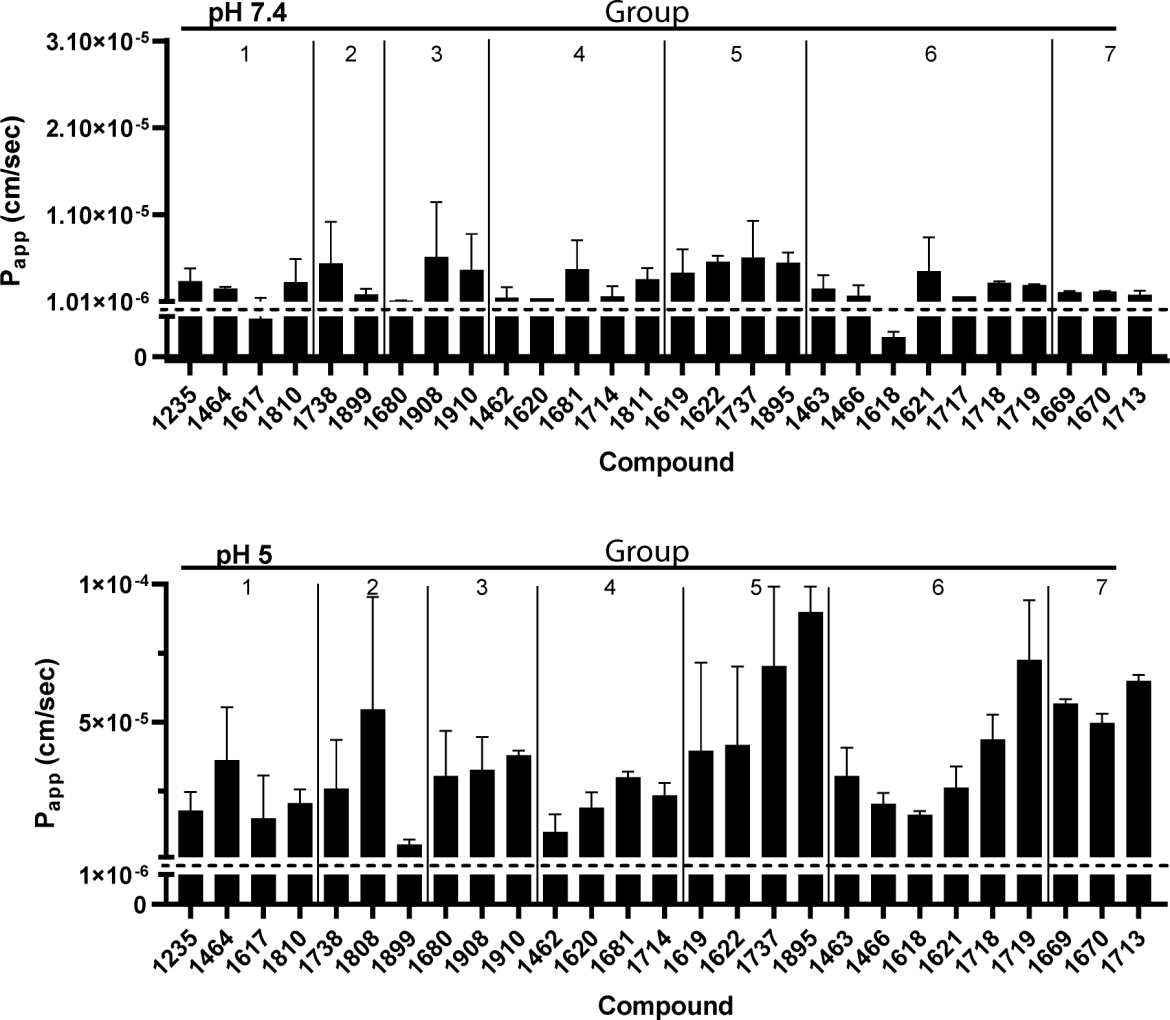
Effect of pH on HPD apparent passive permeability. Compounds with solubility limits >100 µM were tested in the parallel artificial membrane permeability assay (PAMPA) in fluids at pHs that reflect the GI tract: (Top) pH 7.4 (plasma and tissue culture media); (Bottom) pH 5.0 (fed intestinal state). Apparent permeability (P_app_) was determined to be high (≥1 × 10^−6^ cm/s) or low (<1 × 10^−6^ cm/s), as signified by the dashed line. Data are represented as mean ± 1 standard deviation. *N* ≥ 2.

Passive diffusion across the small intestine is the most common route of drug absorption (37, 38). Therefore, the rates of passive diffusion across a cell membrane were assessed for the compounds with solubility limits ≥100 µM in the parallel artificial membrane permeability assay (PAMPA). The compounds were tested in FessIF (pH 5.0) to mimic fed intestinal fluid in the small intestine and pH 7.4 to mimic plasma and tissue culture conditions (Fig. 3; individual values in Table S2). Apparent permeability (P_app_ [cm/s]) of compounds was classified as low (<1 × 10^−6^ cm/s) or high (≥1 × 10^−6^ cm/s) based on an industry-standard cutoff (39). Almost all compounds had high apparent permeability values at both pHs, with the exception that **1617** (Group 1) and **1618** (Group 6) had low P_app_s at pH 7.4. These data imply that most HPDs will have high passive permeability across cellular membranes.

### MDCK-MDR1 permeability

**Fig 4:**
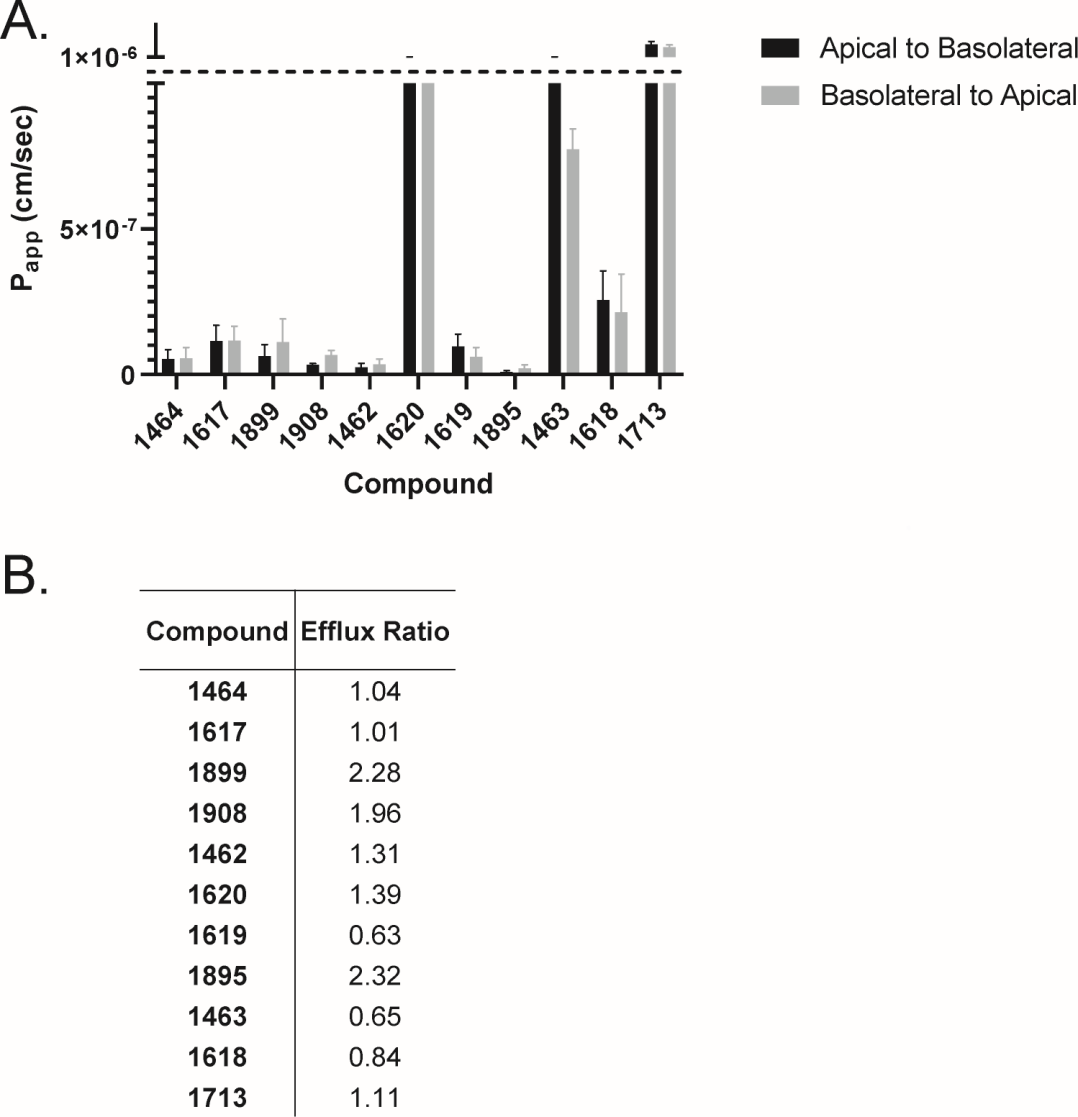
Apparent permeability through MDCK-MDR1 cells. Compounds (10 µM) in HBSS were added to the apical or basolateral side of MDCK-MDR1 cells for 2 h. Samples were retrieved from the recipient well and analyzed by LCMS/MS. (A) Apparent permeability (P_app_ [cm/s]) values were high (>1 × 10^−6^) or low (<1 × 10^−6^), as signified by the dashed line. (B) Active efflux ratios were determined by P_app_ basolateral-apical/apical-basolateral. Data are represented as mean ± 1 standard deviation. *N* ≥ 3.

MDCK-MDR1 cells differentiate into columnar epithelium, form tight junctions in 4–6 days, and highly express p-glycoprotein on the apical surface (40). Therefore, they are useful to evaluate the active transport and paracellular permeability of compounds, as well as assessing whether compounds are likely to be actively effluxed. The apparent permeability (P_app_) and efflux ratio was assessed for 11 structurally diverse compounds: (**1464** and **1617** [Group 1: >1 halogen], **1899** [Group 2: large ether], **1910** [Group 3: 2 R groups], **1462** and **1620** [Group 4: hydrophobic], **1619** and **1895** [Group 5: not halogen], **1463** and **1618** [Group 6: one halogen], and **1713** [Group 7: donating]) (Fig. 4). These compounds were chosen because they were either effective against HBV replication (EC_50_ < 5 µM), had high solubility limits (>100 µM at pH 7.4), or had high rates of apparent permeability by PAMPA (P_app_ ≥1*10^−6^ cm/s).

Cells were grown on transwell inserts with compound added to either the apical or basolateral sides, and compounds were detected in both compartments by LCMS/MS after incubation. Apical to basolateral (A-B) and basolateral to apical (B-A) P_app_s were determined to be high (≥1*10^−6^ cm/s) or low (<1*10^−6^ cm/s), and compounds were determined to be actively effluxed if the B-A/A-B ratio was >2, based on the industry standard cutoffs (39, 41). Compounds **1464**, **1617**, **1899**, **1908**, **1462**, **1619**, **1895**, and **1618** had low P_app_s, while **1620** and **1713** had high P_app_s from A-B and B-A. Interestingly, **1463** had a high P_app_ from A-B and a low P_app_ from B-A, resulting in a low efflux ratio (0.65). **1899** and **1895** are the only compounds that may undergo active efflux with ratios of 2.28 and 2.32, respectively. These data indicate that apparent permeability through MDCK-MDR1 cells is generally low, active efflux is infrequent, and permeability patterns are compound-specific rather than subclass-specific.

### Human RNase H1 inhibition

**Fig 5:**
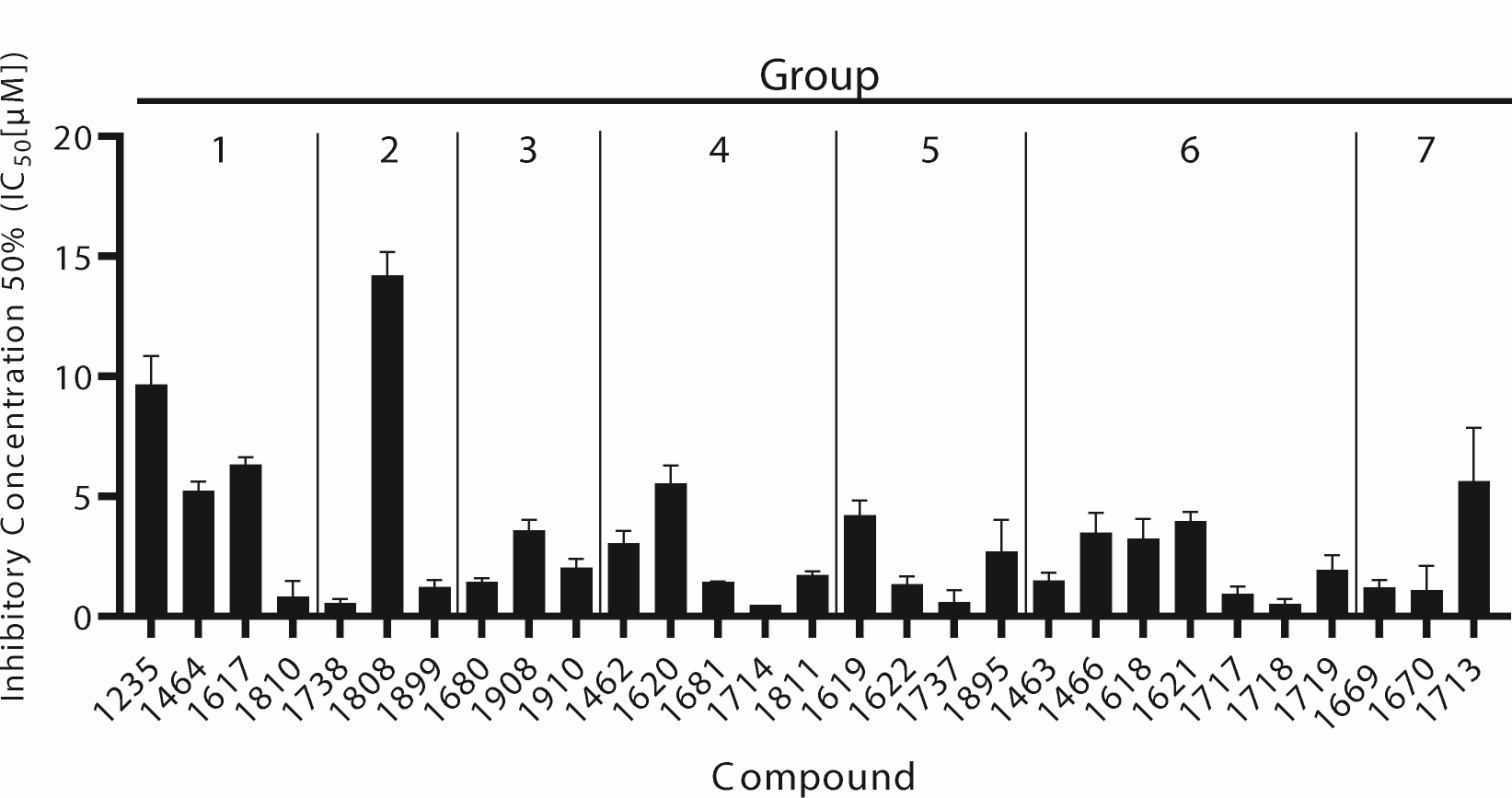
Off-target inhibition of huRNase H1. Inhibitory concentration 50% (IC_50_) values were determined by normalizing the reaction rate of compound at each concentration to the vehicle control (DMSO). Values are reported in µM and represented as mean ± 1 standard deviation. *N* ≥ 3.

The human ribonuclease H1 (huRNase H1) is essential for mitochondrial DNA replication and contributes to the resolution of R-loops in genomic DNA, and it is structurally and enzymatically similar to the HBV RNase H (42, 43). Therefore, we determined the 50% inhibitory concentration (IC_50_) values against huRNase H1 to identify potential off-target effects (Fig. 5). Compounds were screened in a fluorescent biochemical assay, and IC_50_s were determined from the percent activities of the experimental reaction rates normalized to the DMSO vehicle-control reactions. IC_50_ values could not be determined for the HBV RNase H due to difficulties in expressing recombinant enzymes.

Compounds within Group 1 (>1 halogen) had the highest average huRNase H1 IC_50_s (5.51 µM) and compounds within Group 2 (large ether) had the second highest average (5.33 µM); however, **1810** (Group 1) and **1738** (Group 2) had two of the lowest IC_50_ values (0.82 and 0.56 µM, respectively). Importantly, **1464**, **1620**, and **1618**, the most potent compounds against HBV replication, had huRNase H1 IC_50_s ranging from 3.23 to 5.54 µM, which are 27- to 53-fold greater than their HBV RNase H EC_50_s. These data indicate that huRNase H1 IC_50_s are both compound- and subclass-specific, and that huRNase H1 is unlikely to be substantially inhibited at concentrations of the best HPDs anticipated to be used in animal studies or in people.

### Cytochrome P450 inhibition

**Fig 6:**
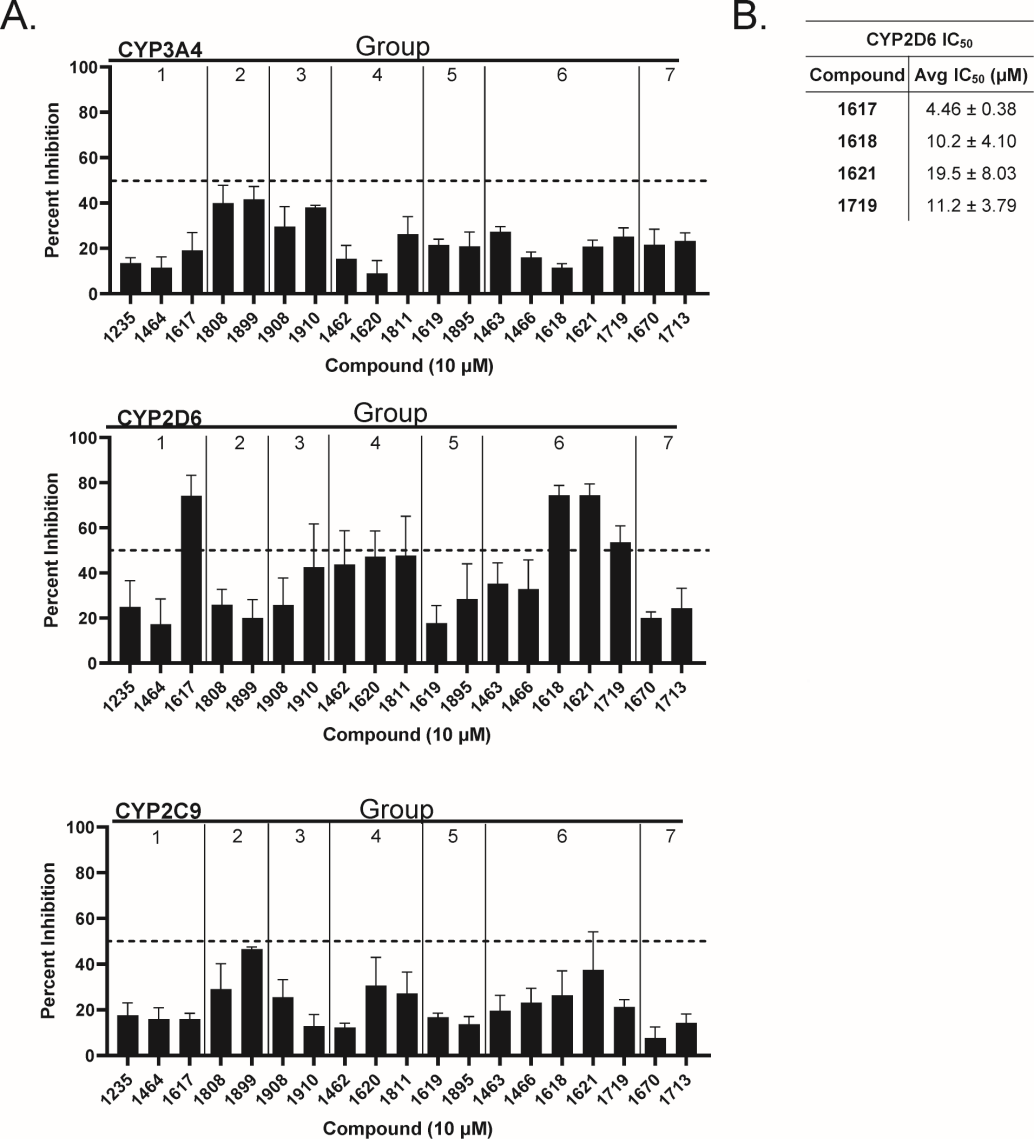
Cytochrome P450 inhibition. Compounds (10 µM) were screened in a biochemical assay against three major CYP450 isoforms. Percent inhibition was determined by subtracting the background of a known CYP450 inhibitor and normalizing it to the reaction rate of a vehicle control (DMSO). (**A)** Percent inhibition by compounds against 3A4, 2D6, and 2C9. (**B).** Inhibitory concentration 50% values for the four compounds that substantially inhibited CYP2D6. Dashed line: 50% inhibition. Data are represented as mean ± 1 standard deviation. *N* ≥ 3.

Drug-drug interactions are commonly attributable to the inhibition of the cytochromes P450 (CYP450), by “perpetrator drugs,” resulting in the decreased metabolism and increased toxicity of other drugs that are substrates for CYP450 (44). As such, we screened compounds against CYP3A4, 2D6, and 2C9 which are responsible for ~65% of CYP450-mediated drug metabolism (45). We qualitatively screened 19 representative compounds from the seven subclasses at 10 µM: **1235**, **1464**, **1617** (Group 1), **1808** and **1899** (Group 2), **1908** and **1910** (Group 3), **1462**, **1620, 1811** (Group 4), **1619, 1895** (Group 5), **1463**, **1466**, **1618**, **1621**, **1719** (Group 6), and **1670**, **1713** (Group 7) (Fig. 6). These compounds were chosen as they inhibit HBV replication at concentrations lower than their huRNase H1 IC_50_ values, have high solubility limits, and are minimally toxic in hepDES19 cells.

Compounds were screened in fluorescent biochemical assays, and percent inhibition was determined by subtracting the positive control reaction rates and normalizing it to DMSO vehicle control reaction rates. None of the compounds substantially inhibited CYP3A4 or 2C9 by >50%; however, **1808** and **1899** in Group 2 inhibited CYP3A4 by ~42%, and **1899** in Group 2 inhibited CYP2C9 by 46%. Compounds **1617**, **1618**, **1621** and **1719** substantially inhibited CYP2D6 by 53–74%. Interestingly, **1617** is in Group 1, while **1618**, **1621**, and **1719** are in Group 6, which suggests the presence of a halogen may increase the potential for CYP2D6 inhibition. Dose-response curves were generated for **1617**, **1618**, **1621**, and **1719** against CYP2D6, and IC_50_ values for these compounds ranged from 4.46 µM (**1617**) to 19.5 µM (**1621**). These data indicate that inhibition of CYP3A4 and 2C9 was minimal by these HPDs, and that CYP2D6 inhibition is more common, with inhibition being both compound- and subclass-specific. The most potent HPD against HBV, **1618**, substantially inhibited CYP2D6, but its selectivity index for CYP inhibition versus its HBV EC_50_ was >150, so it would likely have little impact at doses anticipated to be used if **1618** advances into humans.

### Microsome stability

HPD metabolism by phase I drug metabolizing enzymes was assessed in human liver microsomes by determining compound half-life (*t*_1/2_ [min]) and intrinsic clearance (Cl_int_ [µL/min/mg protein]) following parent compound depletion from a starting concentration of 1 µM over 30 min. We chose 15 representative compounds from the seven subgroups based on either efficacy against HBV replication (EC_50_ < 2 µM) or minimal huRNase H1 (IC_50_ > EC_50_) and CYP450 inhibition (<50%). Compounds chosen were: **1235**, **1464**, **1617** (Group 1), **1808**, **1899** (Group 2), **1908** (Group 3), **1462**, **1620**, **1811** (Group 4), **1619**, **1895** (Group 5), **1463**, **1466**, **1618** (Group 6), and **1713** (Group 7). Compound *t*_1/2_s for 13 experimental compounds were >30 min. Only two compounds had shorter *t*_1/2_s, 23.0 min for **1617** and 29.6 min for **1899** (Table 4). Compounds **1235**, **1620**, and **1618** had low Cl_int_ (µL/min/mg protein) rates (<8.6), and compounds **1617**, **1808**, **1899**, **1908**, **1462**, **1811**, and **1619** had medium Cl_int_ rates (≥ 8.6 and ≤47). The Cl_int_ of **1464**, **1895**, **1463**, and **1466** could not be quantified because the percent remaining at 30 min was greater than the parent compound concentration at 0 min, resulting in a positive slope, which is likely due to noise in the assay. Additionally, **1713** has poor sensitivity by LCMS/MS; therefore, its *t*_1/2_ should be interpreted cautiously.

**TABLE 4:** Compound half-life and intrinsic clearance by phase I drug metabolizing enzymes

Compound	Half-life (min)	Clearance (µL/min/mg)
Ritonavir	23.3	45.2
Warfarin	>30	NQ^*a*^
1235	>30	5.48
1464	>30	NQ
1617	23.0	45.7
1808	>30	10.1
1899	29.6	35.6
1908	>30	11.8
1462	>30	34.8
1620	>30	7.00
1811	NQ	NQ
1619	>30	11.1
1895	>30	NQ
1463	>30	NQ
1466	>30	NQ
1618	>30	5.71
1713^*b*^	>30	NQ

^*a*^ NQ, not quantified.

^*b*^ Poor sensitivity by LCMS/MS.

### Effect of plasma proteins on compound potency

Albumin is the most abundant protein in human serum at 35–50 mg/mL. Many drugs have the propensity to bind to albumin (46), which can result in decreased therapeutic efficacy (47). We assessed the percentage of compounds **1464**, **1617** (Group 1), **1899** (Group 2), **1908** (Group 3), **1462**, **1620** (Group 4), **1619**, **1895** (Group 5), **1463**, **1466**, **1618** (Group 6), and **1713** (Group 7) that bound to albumin by rapid equilibrium dialysis. These compounds were chosen because they had *t*_1/2_s >30 min and for the reasons described in the microsome stability assay, including potent inhibition of HBV replication (EC_50_ < 2 µM) and minimal huRNase H1 or CYP450 inhibition. Compounds were screened by equilibrium dialysis at 10 µM in media conditions reflecting the HBV replication inhibition assay (0.25 mg/mL BSA) and percent bound was determined following LCMS/MS analysis (Table 5). Eleven of 12 HPDs bound plasma proteins at 84–100%, which was greater than the percent of warfarin bound (74%), the high-binding control in this assay. About 13% of **1713** was bound to plasma proteins; however, this compound had low sensitivity by LCMS/MS, so this value should be interpreted cautiously. Only 10% of lamivudine, the low binding control, bound plasma proteins.

**TABLE 5:** *In vitro* percentage of compound bound to plasma proteins

Compound	Percent bound
Warfarin	73.5 ± 1.18
Lamivudine	9.70 ± 11.3
1464	83.7 ± 1.92
1617	100 ± 0.019
1899	87.0 ± 0.974
1908	99.8 ± 0.134
1462	94.9 ± 2.86
1620	85.8 ± 13.2
1619	93.1 ± 2.49
1895	99.5 ± 0.054
1463	96.4 ± 1.93
1466	95.7 ± 1.76
1618	93.5 ± 0.129
1713^*a*^	12.9 ± 12.7

^*a*^ Poor sensitivity by LCMS/MS.

**Fig 7:**
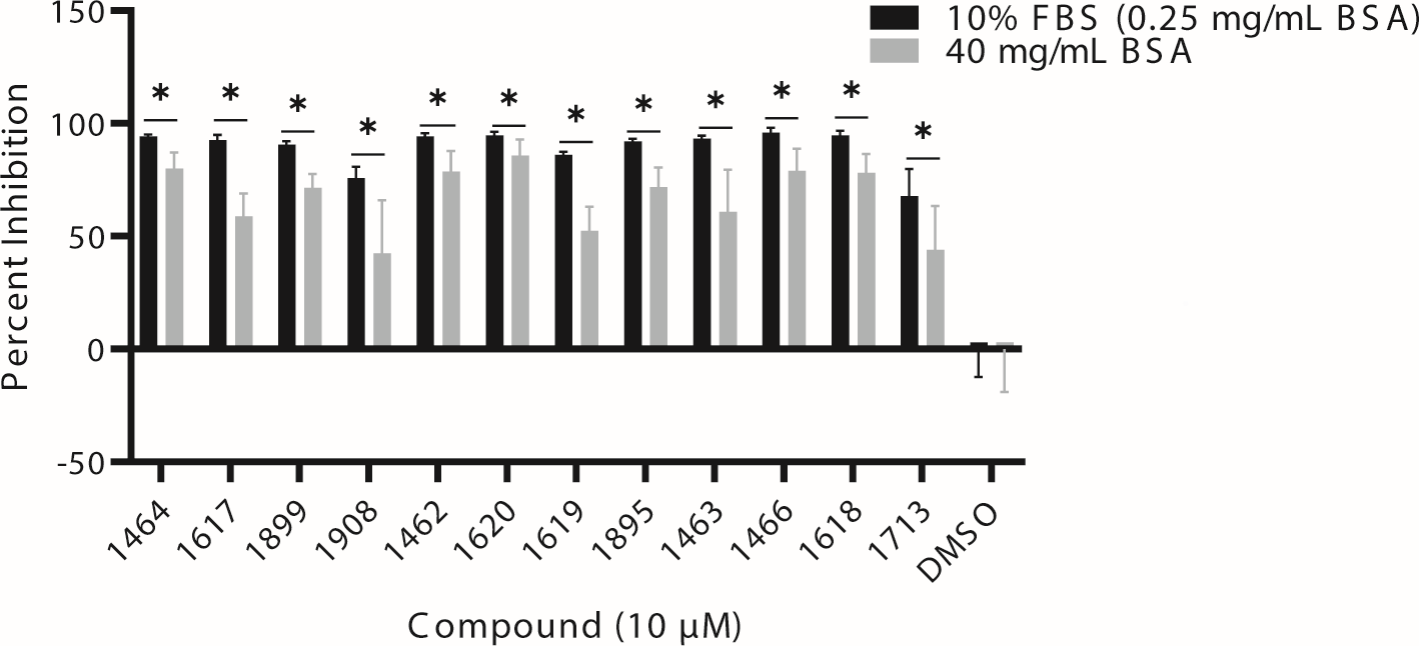
Effect of serum protein concentration on HPD potency. Compounds (10 µM) were added to hepDES19 cells in normal media conditions (10% FBS [0.25 mg/mL BSA]) or media supplemented with BSA to 40 mg/mL. Percent inhibition was determined using qPCR with (+) polarity HBV DNA-specific primers and normalized to DMSO-treated samples. Results are reported as mean ± 1 standard deviation. *N* ≥ 3.

The effect of increased albumin levels on the efficacy of all 12 of these HPDs against HBV replication was assessed following 72 h of compound exposure of hepDES19 cells in media supplemented with BSA to 40 mg/mL (Fig. 7). HBV replication suppression was determined by our strand-preferential qPCR assay at 10 µM, and albumin was determined to affect compound potency if HBV plus polarity DNA suppression was significantly less than values obtained without supplemented BSA. The potency of all 12 HPDs was significantly decreased in the high albumin conditions (*P* < 0.05) (Fig. 7). However, the magnitude of the suppression in this assay was likely larger than would be observed in humans or animal models because the concentration used in this assay averaged 36-fold greater than the compounds EC_50_s (range 2× [**1713**] to 167× [**1618**]), which compresses their efficacy to near 100% in the low albumin conditions. Consequently, the effects of higher albumin are likely only physiologically relevant for **1617**.

### CYP3A4 induction

**Fig 8:**
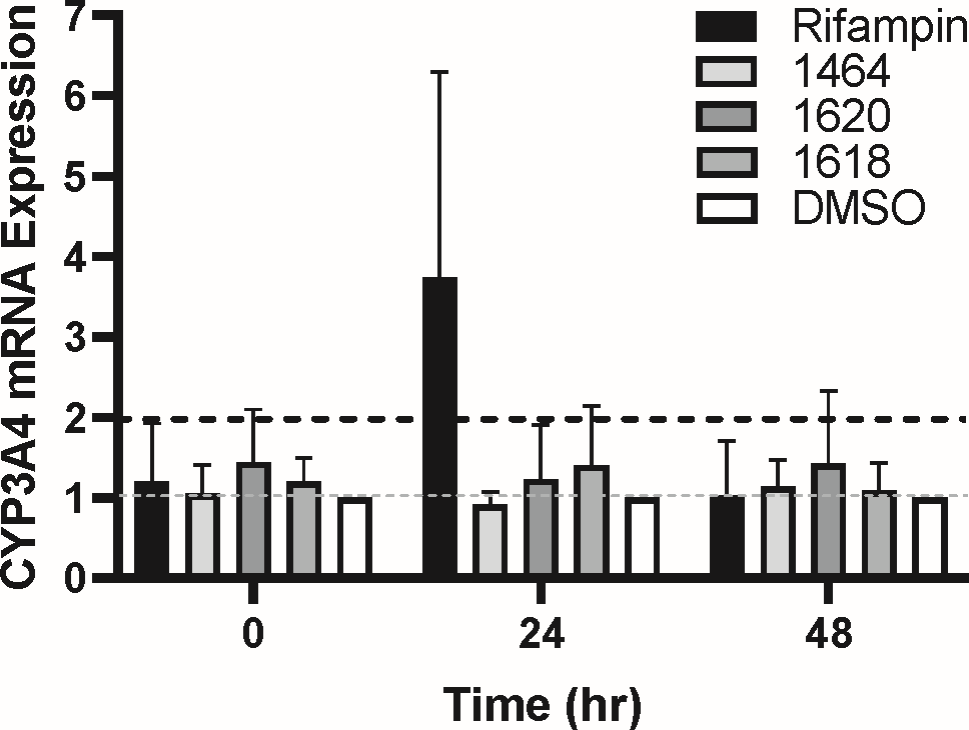
CYP3A4 Induction. Hepatoblastoma cells were treated with 10 µM compound or DMSO, and cells were lysed at 0, 24, and 48 h post compound addition. CYP3A4 expression was measured by RT-qPCR and induction was calculated by normalizing to GAPDH and DMSO. Gray dashed line: basal CYP3A4 expression. Black dashed line: CYP3A4 mRNA expression increases by twofold. *N* > 3.

Drug-drug interactions are also attributable to increase of CYP450 expression or activity, although to a lesser extent than enzymatic inhibition. This often leads to increased metabolism and decreased therapeutic efficacy of a substrate drug (48). We assessed the induction of CYP3A4, as it metabolizes about 60% of all drugs, and thus if its expression is increased, it poses the most risk for adverse drug reactions. The increase of CYP3A4 mRNA expression was assessed following treatment of **1464**, **1618**, and **1620**. These compounds were chosen because they have the highest selectivity indexes (CC_50_/EC_50_) and have the most favorable pharmacological profiles from the previous results. HepG2 cells were treated with 10 µM of experimental compound, rifampin, or 1% DMSO (vehicle) for 0, 24, and 48 h, and CYP3A4 mRNA expression was determined by RT-qPCR. CYP3A4 expression was normalized to both GAPDH- and DMSO-treated wells. A compound was determined to induce CYP3A4 expression if the log fold-change value was ≥2. None of the compounds substantially altered CYP3A4 expression at 24 or 48 h compound treatment; however, the positive control rifampin increased expression as expected (Fig. 8).

## DISCUSSION

We have previously shown that the HPDs are the most effective of the known HBV RNase H inhibitors, with submicromolar half-maximal effective concentrations and minimal toxicity, resulting in selectivity indexes (cytotoxicity/efficacy) >300 (22); however, they lack pharmacological evaluation. Our goal was to analyze the HBV efficacy, cytotoxicity, and evaluate the absorption, metabolism, and distribution parameters of 29 structurally diverse HPDs to guide identification of lead candidate compounds within this chemotype.

There were no apparent cytotoxic trends in hepDES19 cells between the HPD subgroups because although compounds in Group 2 (large ether R group) were on average the most cytotoxic with average CC_50_ values of 50 µM, compounds with CC_50_s >50 µM are considered minimally toxic (Table 3). Our data indicate that cytotoxicity in primary human hepatocytes is minimal and is not reflected by cytotoxicity in hepatoblastoma cells (Table 2), as we have observed previously with two early HPD compounds (21). We hypothesize this discrepancy is because primary hepatocytes are quiescent; therefore, enzymes and proteins that are necessary for metabolism and cell division are downregulated, resulting in a decrease in compound uptake and a decrease in sensitivity to adverse effects caused by rapid cell division.

Efficacy against HBV replication, however, appears to exhibit trends between subgroups (Table 3). Compounds within Group 6 (1 halogen) were the most potent against HBV replication with an average EC_50_ = 0.81 µM, and compounds within Group 1 (>1 halogen) were the second most potent with an average EC_50_ = 1.06 µM. Additionally, compounds within Group 2 (large ether) and Group 3 (two R groups) were the least potent (average EC_50_ >3 µM). Finally, compound **1618** (Group 6), is the most potent HBV RNase H inhibitor identified with an EC_50_ = 0.061 µM, and **1464** (Group 1) is the second most potent inhibitor with an EC_50_ = 0.188 µM. These data indicate that a strong electron-withdrawing substituent on the benzene ring increases compound potency, and that the presence of a larger R group or higher molecular weight (≥400 Da) decreases potency, potentially because the compound cannot efficiently get into cells, is sterically hindered, or it is too large to access the RNase H’s active site for Mg^2+^ coordination. Interestingly, the benzene ring of **1620** (Group 4 [hydrophobic]) has a methyl group but is the third most potent compound identified (EC_50_ = 0.19 µM). Methyl groups are typically weak electron-donating groups; however, this methyl group is at the meta position on the benzene ring, as is the fluorine in **1618**, implying that the location of additional substituents also impacts compound potency.

Absorption across the GI tract is directly proportional to compound solubility and permeability, which are dependent on physiological conditions and a compound’s physicochemical properties, such as molecular weight and lipophilicity (logP) (38). We assessed whether 29 HPDs were likely to precipitate in pHs that reflect the GI tract and plasma (Fig. 2). All compounds in each group had high solubility limits at pH 7.4 (plasma), while **1811** (Group 4 [Hydrophobic]) and **1717** (Group 6 [1 Halogen]) were partially soluble (1–100 µM) at pH 5 (fed intestinal state) and 6.5 (fasted intestinal state), respectively. However, after converting the solubility limits of **1811** and **1717** to the industry standard units of mg/mL, both compounds are freely soluble (100–1,000 mg/mL) (49), and they are unlikely to precipitate at concentrations required to inhibit HBV replication. The high solubility of the compounds is likely driven by their relatively high hydrophilicity.

Compounds that were highly soluble at pH 5 and 7.4 were assessed for their apparent passive permeability (P_app_) in parallel artificial membrane permeability assays (Fig. 3). All compounds had high P_app_s at pH 5, and only **1617** (Group 1) and **1618** (Group 6) had low P_app_s at pH 7.4. The structures of these compounds are similar, in that they both have a fluorine at the meta position on the benzene ring (Fig. S1), suggesting that the location and type of halogen influences passive permeability of HPD compounds. However, these data also indicate that apparent rates of passive permeability do not dictate compound potency because **1617** and **1618** have EC_50_s < 400 nM. Transcellular passive permeability is the most common mode of transport across the GI tract (37); however, paracellular permeability is more likely for polar compounds such as most HPDs. Therefore, we assessed the P_app_s and efflux ratios of 11 structurally diverse HPDs in the MDCK-MDR1 permeability assay (Fig. 4), and only three compounds, **1620**, **1463**, and **1713**, had high P_app_s. We hypothesize the discrepancy between these data and PAMPA is because the MDCK-MDR1 tight junction formation reflects the blood-brain barrier better than the GI tract (50, 51). Additionally, all compounds tested were effective against HBV replication after incubation with compound for 72 h; therefore, it is likely that the 2 h incubation used in the assays is not long enough to assess HPD paracellular permeability. Finally, only **1895** (Group 5) and **1899** (Group 2) are potential p-glycoprotein substrates (efflux ratio >2). P-glycoprotein is a promiscuous substrate binder (52), which explains the lack of trend observed in these data, as the R groups between **1895** and **1899** (Fig. S1) are quite different.

Off-target effects are essential to minimize during lead candidate drug discovery studies; therefore, we determined IC_50_ values for all 29 compounds against human RNase H1, which is structurally and enzymatically similar to HBV RNase H (Fig. 5). The selectivity for HBV plus polarity DNA strand suppression over huRNase H1 activity was <1 for 11 compounds that spanned all seven subgroups, and as such, they were not included in subsequent pharmacological analyses due to the likelihood of huRNase H1-mediated toxicity. Importantly, the selectivity of HBV replication inhibition over huRNase H1 suppression for **1464** and **1617** (Group 1), **1462** and **1620** (Group 4), and **1466** and **1618** (Group 6) was at least 10-fold. Therefore, these compounds are likely to exhibit minimal off-target effects at concentrations required to inhibit HBV replication if moved forward to *in vivo* testing.

The potential for drug-drug interactions rises to 50% for patients taking at least five drugs, and they are primarily attributable to cytochrome P (CYP) 450 inhibition (53). We screened 19 HPDs against CYP3A4, 2D6, and 2C9, which account for ~65% of phase I drug metabolism. While no compounds substantially inhibited (≥50%) CYP3A4 or 2C9, **1808** and **1899** (Group 2) inhibited CYP3A4 by ~41%, and **1899** inhibited CYP2C9 by 49% (Fig. 6). This was anticipated, as CYP2C9 and 3A4 inhibitors are typically larger and lipophilic (54), and **1808** and **1899** have molecular weights ~450 g/mol and cLogP values >4. Importantly, the HBV replication EC_50_ for **1808** and **1899** are 6 and 1.92 µM, respectively. As such, these compounds could potentially cause drug-drug interactions at concentrations that are required to inhibit HBV replication. CYP2D6 was substantially inhibited by **1618**, **1621**, **1719** (Group 6), and **1617** (Group 1) (Fig. 6). There is no correlation between size and lipophilicity of CYP2D6 inhibitors, but they are generally characterized as having a primary or secondary amine, at least one carbonyl, and are heterocyclic (54). The chemical structures of bupropion, fluoxetine, and paroxetine, three well-described CYP2D6 inhibitors, contain at least one halogen, as do **1618**, **1621**, **1719**, and **1617**. Therefore, given the similarities in structure between known CYP2D6 inhibitors and HPDs, it is necessary to take into consideration the compound’s selectivity for HBV over CYP2D6 inhibition when identifying lead candidate compounds. Importantly, the selectivity index of **1618** for HBV replication suppression over CYP inhibition is 166; therefore, it is unlikely to cause off-target effects if dosed in humans.

Human liver microsomes are the best model to assess compound *t*_1/2_ and Cl_int_ in lead candidate identification analyses, as they are amenable to high throughput testing and assess phase I drug metabolism (Table 4). Our data indicate that intrinsic clearance is unlikely to be mediated by phase I drug metabolizing enzymes for 13 of 15 HPDs. Future additional studies will need to be performed to determine the Cl_int_ by phase II drug metabolism for these compounds. The Cl_int_ of **1617** (45.7 µL/min/mg) was similar to the control ritonavir (45.2 µL/min/mg), and as such, is likely to partially be cleared by phase I drug metabolism enzymes. Interestingly, the structures of **1464** and **1617** only differ in the position of one fluorine (Fig. S1); however, **1464** is 3× more potent, which may be explained by its long half-life (>30 min) by phase I drug metabolism. The *t*_1/2_ and Cl_int_ of **1811** could not be determined because the analyte peak traces of **1811** were below the lower limit of detection, despite high sensitivity by LCMS/MS in method development. We hypothesize this discrepancy may be because **1811** is binding to microsomal proteins, and as such **1811** is pelleted with the microsomes and not in the supernatant for analyses. This hypothesis is in accordance with Austin et al., which reports on the nonspecific binding of lipophilic compounds to microsomes (55).

We next assessed whether 12 structurally diverse HPDs bound to albumin, and we determined that 11 compounds bound to albumin at percentages greater than the positive control warfarin (Table 5). There was no general trend in the structure of the compounds that bound to albumin, which was expected because albumin ligands vary greatly in structure and physicochemical properties (56). We next assessed whether compound potency against HBV replication was affected at albumin levels similar to that of human serum, and we determined that the potency of all compounds was significantly but modestly decreased when albumin levels were increased from 0.25 to 40 mg/mL (Fig. 7). Bovine serum albumin and human serum albumin only share 76% sequence homology (57); therefore, follow-up studies to assess the binding of the promising HPDs to human serum proteins will be needed.

Drug-drug interactions are also attributable to induction of CYP450, most notably by increasing CYP3A4 mRNA expression. Therefore, we assessed whether **1464** (Group 1), **1618** (Group 6), and **1620** (Group 4) induced CYP3A4 expression, and we determined that no compound increased CYP3A4 by ≥2-fold (Fig. 8). Therefore, these compounds are unlikely to cause drug-drug interactions by this mechanism at concentrations required to inhibit HBV replication.

Taken together, these results imply that *in vitro* pharmacological parameters of the HPDs are both compound- and subgroup-specific. Additionally, these data indicate that a compound’s pharmacological profile must be wholly examined, as one or two unfavorable parameters do not necessitate disregarding the compound or subgroup from future analyses. However, the HPDs in Group 2 (large ether) and Group 3 (two R groups) should not be pursued as lead candidate compounds due to their poor efficacy and potential for off-target effects. We will continue to synthesize iterations of compounds within Group 1 (>1 halogen) and Group 6 (one halogen), as they are the most potent with minimal off-target effects. Additionally, **1464** (Group 1), **1618** (Group 6), and **1620** (Group 4) will be assessed in mouse pharmacokinetic studies, as the *in vivo* clearance rates and volumes of distribution will guide the ongoing medicinal chemistry campaign. Finally, we identified **1618** (Group 6) and **1620** (Group 4) as lead candidate HPDs and will be testing them for efficacy in an HBV mouse model in future studies because they are potent against HBV replication, have favorable pharmacological parameters similar to drugs on the market, and are unlikely to cause drug-drug interactions at concentrations required to inhibit HBV replication.

## Data Availability

Data and protocols used can be found on Zenodo at the following DOI: 10.5281/zenodo.13947654.

## SUPPLEMENTAL MATERIAL

Figure S1 aac.01455-24-s0001.docx Structures of the 29 compounds used in this study.

Supplemental methods aac.01455-24-s0002.docx Compound synthetic scheme for the previously unpublished compounds used in this study.

Table S1 aac.01455-24-s0003.xlsx Full data table of compound solubility limits.

Table S2 aac.01455-24-s0004.xlsx Full data table of parallel artificial membrane permeability values.

ASM does not own the copyrights to Supplemental Material that may be linked to, or accessed through, an article. The authors have granted ASM a non-exclusive, world-wide license to publish the Supplemental Material files. Please contact the corresponding author directly for reuse.
