# Correction for Woodson et al., “Efficacy and *in vitro* pharmacological assessment of novel *N*-hydroxypyridinediones as hepatitis B virus ribonuclease H inhibitors”

**DOI:** 10.1128/aac.01199-25

**Published:** 2025-11-06

**Authors:** Molly E. Woodson, Holly F. Walden, M. Abdul Mottaleb, Maria Makri, Georgia-Myrto Prifti, Dimitrios Moianos, Vasiliki Pardali, Grigoris Zoidis, John E. Tavis

## AUTHOR CORRECTION

Volume 69, no. 1, e01455-24, 2025, https://doi.org/10.1128/aac.01455-24. Page 9, Table 3: The fourth column should read as shown in this correction. The EC_50_ and CC_50_ values in the original table were correct, but we inadvertently calculated the SI values incorrectly. This error did not materially affect any conclusions within the paper. We apologize for this mistake.

**TABLE 3:** HPD efficacy and cytotoxicity

HepDES19 SI (CC_50_/EC_50_)
Group 1: Electron withdrawing substituents (>1 halogen)
26.3
338
208
54.1
Group 2: Large ether R group
6.86
6.37
35.7
Group 3: 2 R groups bound to central carbon
21.1
28.9
30.0
Group 4: Hydrophobic side chain
112
526
25.2
21.6
45.9
Group 5: Electron withdrawing substituent - not a halogen
87.7
71.4
11.9
91.4
Group 6: Electron withdrawing substituent - one halogen
243
130
1211
151
67.9
40.4
42.1
Group 7: Electron donating substituent
80.6
47.0
17.7

