# Supplementary material for: Efficacy and in vitro pharmacological assessment of novel N-hydroxypyridinediones as hepatitis B virus ribonuclease H inhibitors
Source: Antimicrob Agents Chemother. 2024 Nov 27;69(1):e01455-24. doi: 10.1128/aac.01455-24 (PMC11784145; doi:10.1128/aac.01455-24)
Supplement: Figure S1 — Structures of the 29 compounds used in this study. [file aac.01455-24-s0001.docx]

| **Group 1: Electron withdrawing substituent (>1 halogen)** |
| --- |
| **1235**  **1464**  **1617**  **1810** |
| **Group 2: Large ether R group** |
| **1899**  **1808**  **1738** |
| **Group 3: 2 R groups bound to central carbon** |
| **1908**  **1910**  **1680** |
| **Group 4: Hydrophobic side chain** |
| **1714**  **1681**  **1620**  **1811**  **1462** |

| **Group 5: Electron withdrawing substituent (not halogen)** |
| --- |
| **1895**  **1737**  **1622**  **1619** |
| **Group 6: Electron withdrawing substituent (1 halogen)** |
| **1618**  **1463**  **1466**  **1621**  **1719**  **1718**  **1717** |
| **Group 7: Electron donating substituent** |
| **1669**  **1670**  **1713** |

**Supplementary Figure S1. Structures of 29 HPDs used in this study.**
