# Supplementary material for: Efficacy and in vitro pharmacological assessment of novel N-hydroxypyridinediones as hepatitis B virus ribonuclease H inhibitors
Source: Antimicrob Agents Chemother. 2024 Nov 27;69(1):e01455-24. doi: 10.1128/aac.01455-24 (PMC11784145; doi:10.1128/aac.01455-24)
Supplement: Supplemental methods — Compound synthetic scheme for the previously unpublished compounds used in this study. [file aac.01455-24-s0002.docx]

**Appendix: Compound Synthesis**

**Materials and Methods**

*Chemistry – General Part*

All reagents and starting materials were purchased from commercial suppliers and used without further purification. Anhydrous CH_2_Cl_2_ was obtained by distillation from calcium hydride under argon. Anhydrous THF was freshly distilled from Na and benzophenone ketyl. All non-aqueous reactions were performed under an inert atmosphere of argon. Concentrated refers to the removal of solvent with a rotary evaporator at normal water aspirator pressure, followed by further evacuation on a high-vacuum line. Thin-layer chromatography was performed using silica gel 60 Å precoated aluminum or glass-backed plates (0.25 mm thickness) with fluorescent indicators. Developed TLC plates were visualized with UV light (254 nm), iodine vapors, or anisaldehyde staining solution. The chromatographic purification of the products was carried out using Fluka silica gel 60 for preparative column chromatography (particle size 40–63 μm). Melting points were determined using a Büchi 530 device and presented without using corrections. NMR spectra were obtained in CDCl_3_ or DMSO-*d*_6_ at 25 °C on a Bruker Avance DRX 600, 500, 400 or 250 MHz spectrometer. The measured chemical shifts are reported in *δ* (ppm), and the residual solvent signal was used as the internal calibration standard (CDCl_3_): ^1^H = 7.26 ppm, ^13^C = 77.18 ppm); (DMSO-*d*_6_): ^1^H = 2.50 ppm, ^13^C = 39.51 ppm. ^13^C-NMR spectra were obtained with complete proton decoupling. Data of NMR spectra were recorded as follows: s = singlet, d = doublet, t = triplet, m = multiplet, dd = doublet of doublets, td = triplet of doublets, tt = triplet of triplets and br = broad signal. The coupling constant *J* is reported in hertz (Hz). ^1^H- and ^13^C-NMR peaks were assigned based on the combined analysis of a series of ^1^H-^1^H (COSY) and ^1^H-^13^C (HSQC, HMBC) correlation spectra.

**Scheme 1.** Synthesis of tested compounds.

**Synthesis of *O*-Substituted *N*-Hydroxyphthalimides**

General procedure:

To a solution of *N*-hydroxyphthalimide (500.0 mg, 3.07 mmol, 1 equiv.) in anhydrous DMF (3 mL), NaH 60% w/w (1.25 equiv.) is added at 0 °C. The mixture is stirred at rt for 30 min. Thereafter, the appropriate halogenide (1.5 equiv.) is added and the reaction is stirred at rt overnight. Then, water is added and a solid precipitate is formed. The precipitate is filtered under vacuum and washed with water and a solution of n-pentane/Et_2_O 7:3. The solid is dried over P_2_O_5_, to afford the desired product.

The compound 2-((4-(trifluoromethyl)benzyl)oxy)isoindoline-1,3-dione (**1**) was synthesized from 1-(bromomethyl)-4-(trifluoromethyl)benzene according to the general procedure. White solid (2.6 g, 66%) (1).

The compound 2-((2,4-difluorobenzyl)oxy)isoindoline-1,3-dione (**2**) was synthesized from 1-(bromomethyl)-2,4-difluorobenzene according to the general procedure. White solid (607,3 mg, 86 %). (35830169). ^1^H NMR (400 MHz,CDCl_3_ ) δ 7.77−7.75 (m, 4H), 7.60−7.52 (m, 3H), 5.27 (s, 2H).

The compound 2-((3,4-difluorobenzyl)oxy)isoindoline-1,3-dione (**3**) was synthesized from 1-(bromomethyl)-3,4-difluorobenzene according to the general procedure. Pale pink solid (684.6 mg, 97 %). R*_f_* 0.53 (CH_2_Cl_2_), mp: 155-157 ^o^C. ^1^H NMR (400 MHz, DMSO) δ 7.86 (s, 4H, Αr- Pthal), 7.64 (ddd, *J* = 10.3, 8.0, 2.0 Hz, 1H, Ar), 7.52 – 7.33 (m, 2H, Ar), 5.17 (s, 2H, OCH_2_). ^13^C NMR (101 MHz, DMSO) δ 163.07 (C_1_, C_3_), 150.94 (C_3’_),134.84 (C_4’_), 132.36 (C_1’_), 128.48 (C_5,_ C_6_), 126.83 (C_3a_, C_7a_), 123.31 (C_6’_), 118.78 (C_4_,C_7_), 118.61 (C_5’_), 117.61 (C_2’_), 77.81 (OCH_2_).

The compound 2-((2,5-difluorobenzyl)oxy)isoindoline-1,3-dione (**4**) was synthesized from 1-(bromomethyl)-2,5-difluorobenzene according to the general procedure. White solid (563.8mg, 80 %). R*_f_* 0.53 (CH_2_Cl_2_), mp: 127-129 ^o^C. ^1^H NMR (400 MHz, DMSO) δ 7.85 (s, 4H, Ar- Pthal), 7.49 (ddt, *J* = 8.3, 6.6, 1.8 Hz, 1H, Ar), 7.30 (ddt, *J* = 10.4, 6.8, 2.8 Hz, 2H, Ar), 5.22 (d, *J* = 1.3 Hz, 2H, OCH_2_). ^13^C NMR (101 MHz, CDCl_3_) δ 163.3 (C_1_, C_3_), 135.8 (C_1’_), 135.3 (C_5’_), 134.5 (C_2’_), 132.4 (C_3a_, C_7a_), 120.5 (C_5_, C_6_), 129.5 (C_3’_), 128.7 (C_4’_), 123.6 (C_4_,C_7_), 75.7 (OCH_2_).

The compound 2-((4-(benzyloxy)benzyl)oxy)isoindoline-1,3-dione (**5**) was synthesized from 1-(benzyloxy)-4-(chloromethyl)benzene according to the general procedure. White solid (31.4 mg, 63 %). R*_f_* 0.63 (CH_2_Cl_2_), mp: 170-172 ^o^C. ^1^H NMR (600 MHz, DMSO) δ 7.85 (s, 4H, Ar- Pthal), 7.44 (tt, *J* = 9.5, 2.3 Hz, 4H, Ar), 7.41 – 7.37 (m, 2H, Ar), 7.35 – 7.30 (m, 1H, Ar), 7.04 – 7.01 (m, 2H, Ar), 5.12 (s, 2H, OCH_2_), 5.09 (s, 2H, OCH_2_). ^13^C NMR (100 MHz, DMSO) d: 161.0 (C_1_, C_3_), 157.9 (C_4’_), 136.7 (C_1’’_), 132.2 (C_5_, C_6_), 132.0 (C_3a_, C_7a_), 129.3 (C_2’_, C_6’_), 128.9 (C_3’’_, C_5’’_), 128.8 (C_1’_), 127.6 (C_4’’_), 127.1 (C_2’’_, C_6’’_), 123.7 (C_4_, C_7_), 114.5 (C_3’_, C_5’_), 78.3 (OCH_2_), 70.8 (OCH_2_). Anal. Calcd for C_22_H_17_NO_4_: C, 73.53; N, 3.90; H, 4.77. Found: C, 73.55; N, 3.92; H, 4.80.

The compound 2-((4-((4-(tert-butyl)benzyl)oxy)benzyl)oxy)isoindoline-1,3-dione (**6**) was synthesized from 1-(*tert*-butyl)-4-((4-(chloromethyl)phenoxy)methyl)benzene according to the general procedure. Off-white solid (200 mg, 66 %). R*_f_* 0.63 (CH_2_Cl_2_), mp: 168-170 ^o^C. ^1^H NMR (400 MHz, DMSO) δ 7.85 (s, 4H, Ar- Pthal), 7.51 (d, *J* = 2.2 Hz, 1H, Ar), 7.44 (d, *J* = 2.1 Hz, 1H, Ar), 7.43 – 7.36 (m, 4H, Ar), 7.06 – 7.00 (m, 2H, Ar), 5.14 (s, 2H, OCH_2_), 5.08 (s, 2H, OCH_2_). ^13^C NMR (101 MHz, DMSO) δ 163.80 (C_1_, C_3_), 159.66 (C_4’_), 150.98 (C_4’’_), 148.04 (C_2’’_), 135.44 (C_5_, C_6_), 134.50 (C_3a_, C_7a_), 132.12 (C_2’_, C_6’_), 129.15 (C_1’_), 128.28 (C_6’’_), 126.94 (C_3’’_, C_5’’_), 125.81 (C_1’’_), 123.88 (C_4_, C_7_), 115.26 (C_3’_, C_5’_), 79.48 (OCH_2_), 69.64 (OCH_2_), 34.93 (C-CH_3_) , 31.76 (CH_3_). Anal. Calcd for C_22_H_16_ClNO_4_: C, 75.16; N, 3.37; H, 6.07. Found: C, 75.17; N, 3.39; H, 6.09.

The compound 2-(naphthalen-2-ylmethoxy)isoindoline-1,3-dione (**7**) was synthesized from 2-((4-(chloromethyl)phenoxy)methyl)naphthalene according to the general procedure. White solid (400 mg, 54 %). R*_f_* 0.53 (CH_2_Cl_2_), mp: 130-132 ^o^C. ^1^H NMR (400 MHz, DMSO) δ 8.02 (s, 1H, Αr), 8.00 – 7.91 (m, 3H, Ar), 7.85 (s, 4H, Ar- Pthal), 7.69 (dd, *J* = 8.5, 1.7 Hz, 1H, Ar), 7.55 (qd, *J* = 6.9, 3.4 Hz, 2H, Ar), 5.34 (s, 2H, OCH_2_). ^13^C NMR (101 MHz, CDCl_3_) δ 163.3 (C_1_, C_3_), 135.8 (C_1’_), 135.3 (C_3’_), 134.5 (C_5_, C_6_), 132.4 (C_3a_, C_7a_), 131.8 (C_4’_), 129.5 (C_6’_), 128.7 (C_10’_), 128.6 (C_7’_), 127.6 (C_5’_), 127.1 (C_2’_), 126.2 (C_8’_), 125.4 (C_9’_), 124.2 (C_4_, C_7_), 75.7 (OCH_2_). Anal. Calcd for C_19_H_13_NO_3_: C, 75.24; N, 4.62; H, 4.32. Found: C, 75.26; N, 4.66; H, 4.35.

The compound 2-((4-methylbenzyl)oxy)isoindoline-1,3-dione (**8**) was synthesized from 1-(bromomethyl)-4-methylbenzene according to the general procedure. White solid (600,7 mg, 92 %). ^1^H NMR (600 MHz, CDCl_3_) δ 7.80 (dt, J = 7.0, 3.5 Hz, 2H), 7.76 – 7.70 (m, 2H), 7.42 (d, J = 7.9 Hz, 2H), 7.18 (d, J = 7.8 Hz, 2H), 5.17 (s, 2H), 2.35 (s, 3H) (2).

The compound 2-((3-methylbenzyl)oxy)isoindoline-1,3-dione (**9**) was synthesized from 1-(bromomethyl)-3-methylbenzene according to the general procedure. White solid ((534.5 mg, 66 %). R*_f_* 0.53 (CH_2_Cl_2_), mp: 131-133 ^o^C; ^1^H NMR (600 MHz, CDCl_3_) δ 7.82 (dd, *J* = 5.4, 3.1 Hz, 2H, Ar-Pthalimide), 7.73 (dd, *J* = 5.5, 3.0 Hz, 2H, Ar-Pthalimide), 7.37 – 7.31 (m, 2H, Ar), 7.27 (d, *J* = 7.5 Hz, 1H, Ar), 7.18 (d, *J* = 7.6 Hz, 1H, Ar), 5.18 (s, 2H, OCH_2_), 2.36 (s, 3H, CH_3_). ^13^C NMR (101 MHz, CDCl_3_) δ 163.62 (C_1_, C_3_) 138.40 (C_1’_), 134.54 ( C_3’_), 133.67 ( C_7a_,C_3a_), 130.70 (C_7_, C_4_), 130.22 (C_6_, C_5_), 129.02 (C_2’_), 128.56 (C_5’_), 127.03 ( C_4’_), 123.61(C_7_, C_4_), 80.11 (1’-CH2), 21.42 ( 3’-CH_3_). Anal. Calcd for C_16_H_13_NO_3_: C, 71.90; H, 4.90; N, 5.24. Found: C, 71.94; H, 4.93; N, 5.26.

The compound 2-((2-methylbenzyl)oxy)isoindoline-1,3-dione (**10**) was synthesized from 1-(bromomethyl)-2-methylbenzene according to the general procedure. White solid (753.6 mg, 93 %). R*_f_* 0.61 (CH_2_Cl_2_); mp: 129-131 ^o^C. ^1^H NMR (400 MHz, CDCl_3_) δ 7.83 – 7.78 (m, 2H, Ar-Pthalimide), 7.76 – 7.70 (m, 2H, Ar-Pthalimide), 7.32 (ddd, *J* = 37.0, 7.3, 1.4 Hz, 2H, Ar), 7.25 – 7.12 (m, 2H, Ar), 5.24 (s, 2H, OCH_2_), 2.61 (s, 3H, CH_3_). ^13^C NMR (101 MHz, CDCl_3_) δ 163.62 (C_1_, C_3_), 139.23 (C_1’_), 134.51 (C_2’_), 131.79 ( C_7a_, C_3a_), 131.42 (C_6_, C_5_), 130.65 (C_5’_), 129.86 (C_4’_), 129.00 (C_3’_), 125.96 (C_7_, C_4_), 123.55 (C_5’_), 78.16 (9-CH_2_), 19.13 (2’-CH_3_). Anal. Calcd for C_16_H_13_NO_3_: C, 71.90; H, 4.90; N, 5.24. Found: C, 71.93; H, 4.94; N, 5.28.

The compound 2-(naphthalen-2-ylmethoxy)isoindoline-1,3-dione (**11**) was synthesized from 2-(bromomethyl)naphthalene according to the general procedure. White solid (400mg, 53.7%). R*_f_* 0.53 (CH_2_Cl_2_); mp: 130-132 ^o^C. ^1^H NMR (400 MHz, DMSO) δ 8.02 (s, 1H, Αr), 8.00 – 7.91 (m, 3H, Ar), 7.85 (s, 4H, Ar- Pthal), 7.69 (dd, *J* = 8.5, 1.7 Hz, 1H, Ar), 7.55 (qd, *J* = 6.9, 3.4 Hz, 2H, Ar), 5.34 (s, 2H, OCH_2_). ^13^C NMR (101 MHz, CDCl_3_) δ 163.3 (C_1_, C_3_), 135.8 (C_1’_), 135.3 (C_3’_), 134.5 (C_5_, C_6_), 132.4 (C_3a_, C_7a_), 131.8 (C_4’_), 129.5 (C_6’_), 128.7 (C_10’_), 128.6 (C_7’_), 127.6 (C_5’_), 127.1 (C_2’_), 126.2 (C_8’_), 125.4 (C_9’_), 124.2 (C_4_, C_7_), 75.7 (OCH_2_). Anal. Calcd for C_19_H_13_NO_3_: C, 75.24; N, 4.62; H, 4.32. Found: C, 75.26; N, 4.66; H, 4.35.

The compound 2-((3-fluorobenzyl)oxy)isoindoline-1,3-dione (**12**) was synthesized from 1-(bromomethyl)-3-fluorobenzene according to the general procedure. White solid (579.0mg, 87%). R*_f_* 0.53 (CH_2_Cl_2_); mp: 129-131 ^o^C. ^1^H NMR (600 MHz, CDCl_3_) δ 7.84 – 7.80 (m, 2H, Ar- Pthal), 7.74 (dd, *J* = 5.5, 3.1 Hz, 2H, Ar- Pthal), 7.39 – 7.28 (m, 2H, Ar), 7.07 (tt, *J* = 8.1, 1.9 Hz, 2H, Ar), 5.20 (s, 2H, OCH_2_). ^13^C NMR (100 MHz, CDCl3) d: 164.6 (C_3’_), 162.1 (C_1’_), 163.4 (C_1_, C_3_), 134.5 (C_3a_, C_7a_), 131.9 (C_5_, C_6_), 129.6 (C_5’_), 128.8 (C_4_, C_7_), 123.5 (C_6’_), 115.6 (C_2’_, C_4’_), 79.0 (OCH_2_). Anal. Calcd for C_15_H_10_FNO_3_: C, 66.42; N, 5.16; H, 3.72. Found: C, 66.44; N, 5.19; H, 3.74.

The compound 2-((3-bromobenzyl)oxy)isoindoline-1,3-dione (**13**) was synthesized from 1-bromo-3-(bromomethyl)benzene according to the general procedure. White solid (534.5 mg, 66 %). R*_f_* 0.62 (CH_2_Cl_2_); mp: 143-145 ^o^C. ^1^H NMR (600 MHz, CDCl_3_) δ 7.83 (dd, *J* = 5.4, 3.1 Hz, 2H, Ar-Pthalimide), 7.75 (dd, *J* = 5.5, 3.1 Hz, 2H, Ar-Pthalimide), 7.70 (t, *J* = 1.9 Hz, 1H, Ar), 7.53 – 7.48 (m, 2H, Ar), 7.27 (d, *J* = 7.8 Hz, 1H, Ar), 5.17 (s, 2H, OCH_2_). ^13^C NMR (125 MHz, CDCl_3_) δ 163.47 (C_1_, C_3_), 142.66 (C_1’_), 134.83 (C_2’_), 133.84 (C_7a_, C_3a_), 128.66 (C_6_, C_5_), 127.75 (C_4’_), 127.18 (C_5’_), 127.02 (C_6’_), 123.69 (C_7_, C_4_), 123.31 (C_3’_), 80.15 (1’-CH_2_). Anal. Calcd for C_15_H_10_BrNO_3_: C, 54.24; H, 3.03; N, 4.22. Found: C, 54.28; H, 3.07; N, 4.24.

The compound 2-((4-bromobenzyl)oxy)isoindoline-1,3-dione (**14**) was synthesized from 1-bromo-4-(bromomethyl)benzene according to the general procedure. White solid (710.1 mg, 87 %). R*_f_* 0.57 (CH_2_Cl_2_); mp: 134-136 ^o^C. ^1^H NMR (600 MHz, CDCl_3_) δ 7.82 (dd, *J* = 5.4, 3.1 Hz, 2H, Ar-Pthalimide), 7.74 (dd, *J* = 5.5, 3.0 Hz, 2H, Ar-Pthalimide), 7.51 (d, *J* = 8.4 Hz, 2H, Ar), 7.42 (d, *J* = 8.4 Hz, 2H, Ar), 5.17 (s, 2H, OCH_2_). ^13^C NMR (101 MHz, CDCl_3_) δ 163.51 (C_1_, C_3_), 134.61 (C_2’_) , 132.06 (C_5’_, C_3’_), 131.86 (C_7a_, C_3a_), 131.48 (C_6_, C_5_), 128.95 (C_6’_, C_2’_), 123.69 (C_4’_) ,123.65 (C_7_, C_4_), 79.07 (1’-CH_2_). Anal. Calcd for C_15_H_10_BrNO_3_: C, 54.24; H, 3.03; N, 4.22. Found: C, 54.27; H, 3.06; N, 4.25.

The compound 2-((2-bromobenzyl)oxy)isoindoline-1,3-dione (**15**) was synthesized from 1-bromo-2-(bromomethyl)benzene according to the general procedure. White solid (753.6 mg, 93 %). R*_f_* 0.54 (CH_2_Cl_2_); mp: 159–161 ^o^C. ^1^H NMR (600 MHz, CDCl_3_) δ 7.82 (dd, *J* = 5.4, 3.1 Hz, 2H, Ar-Pthalimide), 7.74 (dd, *J* = 5.5, 3.0 Hz, 2H, Ar-Pthalimide), 7.62 (ddd, *J* = 44.3, 7.8, 1.5 Hz, 2H, Ar), 7.35 (td, *J* = 7.5, 1.2 Hz, 1H, Ar), 7.23 (td, *J* = 7.7, 1.7 Hz, 1H, Ar), 5.36 (s, 2H, OCH_2_). ^13^C NMR (101 MHz, CDCl_3_): δ 163.3 (C_1_, C_3_), 134.5 (C_1’_), 133.7 (C_6’_), 132.9 (C_7a_, C_3a_), 131.7 (C_6_, C_5_), 130.7 (C_3’_), 128.9 (C_4’_), 127.6 (C_2’_), 124.5 (C_5’_), 123.5 (C_7_, C_4_), 78.7 (1’-CH_2_). Anal. Calcd for C_15_H_10_BrNO_3_: C, 54.24; H, 3.03; N, 4.22. Found: C, 54.28; H, 3.05; N, 4.26.

The compound 2-((3-chlorobenzyl)oxy)isoindoline-1,3-dione (**16**) was synthesized from 1-chloro-3-(bromomethyl)benzene according to the general procedure. White solid (601.8 mg, 85 %). R*_f_* 0.70 (CH_2_Cl_2_); mp: 104-106 ^o^C. ^1^H NMR (600 MHz, CDCl_3_) δ 7.83 (dd, *J* = 5.4, 3.1 Hz, 2H, Ar-Pthalimide), 7.75 (dd, *J* = 5.5, 3.0 Hz, 2H, Ar-Pthalimide), 7.58 – 7.27 (m, 4H, Ar), 5.18 (s, 2H, OCH_2_). ^13^C NMR (101 MHz, CDCl_3_) δ 163.56 (C_1_, C_3_), 135.80 (C_3’_), 134.70 (C_7a_, C_3a_), 134.55 (C_6_, C_5_), 130.01 (C_5’_), 129.89 (C_4’_), 129.61 (C_2’_), 124.54 (C_6’_), 120.12 (C_7_, C_4_), 82.75 (1’-CH_2_). Anal. Calcd for C_15_H_10_ClNO_3_: C, 62.62; H, 3.50; N, 4.87. Found: C, 62.66; H, 3.54; N, 4.91.

To a solution of N-hydroxyphthalimide (200 mg, 1.23 mmol, 1 equiv.) in dry THF (10 mL), 1-(hydroxymethyl)-3,4-dimethoxybenzene (1.84 mmol, 1.5 equiv.) and PPh_3_ (353.7 mg, 1.35 mmol, 1.1 equiv.) are added. Then, DIAD (265 μL, 1.35 mmol, 1.1 equiv.) is added dropwise at 0 ◦C. The reaction is stirred at rt for 48 h. The reaction mixture is extracted from EtOAc (3 x 50 mL), and the combined organic phases are washed with brine, dried over anh. Na_2_SO_4_, filtered, and concentrated. The resulting crude mixture is purified by crystallization (MeOH) to give 2-((3,4-dimethoxybenzyl)oxy)isoindoline-1,3-dione (**17**) as a white solid (164.9 mg, 45 %). R*_f_* 0.5 (AcOEt); mp: 143-145 ^o^C. ^1^H NMR (400 MHz, CDCl_3_) δ 7.83 – 7.77 (m, 2H, Ar-Pthalimide), 7.75 – 7.69 (m, 2H, Ar-Pthalimide), 7.13 (d, *J* = 2.0 Hz, 1H, Ar), 7.02 (dd, *J* = 8.2, 2.0 Hz, 1H, Ar), 6.82 (d, *J* = 8.2 Hz, 1H, Ar), 5.17 (s, 2H, OCH_2_), 3.91 (s, 3H, OCH_3_), 3.87 (s, 3H, OCH_3_). ^13^C NMR (100 MHz, CDCl_3_) *δ*: 163.5 (C_1_, C_3_), 149.8 (C_3’_), 148.9 (C_4’_), 134.4 (C_1’_), 128.8 (C_7a_, C_3a_), 126.1 (C_6_, C_5_), 123.4 (C_4’_), 122.7 (C_7_, C_4_), 112.7 (C_2’_), 110.6 (C_5’_), 79.7 (1’-CH_2_), 55.9 (3’-OCH_3_), 55.8 (4’-OCH_3_). Anal. Calcd for C_17_H_15_NO_5_: C, 65.17; H, 4.83; N, 4.47. Found: C, 65.21; H, 4.87; N, 4.86.

**General Method for the synthesis of benzylphenylethers *via* Williamson** **reaction**

To a solution of 4-hydroxy benzyl alcohol (300 mg, 2.42 mmol, 1 eq) in anhydrous acetonitrile (5.5 mL) is added Cs_2_CO_3_ (1.57 g, 4.83 mmol) under cooling and allowed to stir at RT for 30 min. Then, the benzyl alkyl halide (2.42 mmol, 1 eq) is added and the reaction is left under argon atmosphere and stirring at 82 °C for 20 hours (overnight). The reaction mixture is filtered and washed with MeCN. The filtrate is collected, concentrated and the desired product is obtained pure in the form of a light colored solid. It is used in the next step without further purification.

The compound (4-(benzyloxy)phenyl)methanol (**17**) was synthesized according to the general procedure. White solid (517.78mg, 99%). R*_f_*  0.13 (hexane/AcOEt 2:1); mp: 84-86 ^o^C. ^1^H NMR (600 MHz, DMSO) δ 7.46 – 7.43 (m, 2H, Ar), 7.41 – 7.37 (m, 2H, Ar), 7.35 – 7.31 (m, 1H, Ar), 7.24 – 7.21 (m, 2H, Ar), 6.98 – 6.95 (m, 2H, Ar), 5.10 (s, 2H, OCH_2_), 4.41 (s, 2H, OCH_2_). ^13^C NMR (75 MHz, DMSO) δ 157.9 (C_4_), 136.7 (C_1’_), 133.5 (C_1_), 129.3 (C_2_, C_6_), 128.9 (C_3’_, C_5’_), 127.6 (C_4’_), 127.1 (C_2’_, C_6’_), 114.5 (C_3_, C_5_), 70.8 (OCH_2_), 64.7 (OCH_2_). Anal. Calcd for C_14_H_14_O_2_: C, 78.48; H, 6.59. Found: C, 78.54; H, 6.61.

The compound (4-((4-(tert-butyl)benzyl)oxy)phenyl)methanol (**18**) was synthesized according to the general procedure. White solid (653.37mg, 99%). R*_f_*  0.13 (hexane/AcOEt 2:1); mp: 88-90 ^o^C. ^1^H NMR (400 MHz, DMSO) δ 7.45 – 7.32 (m, 4H, Ar), 7.27 – 7.18 (m, 2H, Ar), 6.99 – 6.92 (m, 2H, Ar), 5.04 (s, 2H, OCH_2_), 4.42 (d, *J* = 17.1 Hz, 2H, OCH_2_), 1.27 (s, 9H, t-Bu). ^13^C NMR (75 MHz, DMSO) δ 157.9 (C_4_), 150.2 (C_4’_), 133.6 (C_1’_), 133.5 (C_1_), 129.3 (C_2,_ C_6_), 126.7 (C_2’_, C_6’_), 125.2 (C_3’,_ C_5’_), 114.5 (C_3_, C_5_), 70.8 (OCH_2_), 64.7 (OCH_2_), 34.2 (C- t- Bu), 31.3 (t- Bu). Anal. Calcd for C_18_H_22_O_2_: C, 79.96; H, 8.20. Found: C, 79.99; H, 8.21.

**General Method for the synthesis of benzyl chlorides**

A solution of the appropriate substituted benzyl alcohol (2.42 mmol, 1 eq) in thionyl chloride (4.73 mL, 65.25 mmol) (acting as both solvent and chlorination reagent) is allowed to reflux at 76 °C for 1 h. The reaction mixture is concentrated, and a beige solid is obtained. The solid is washed with hexane and filtered. It is used in the next step without further purification.

The compound 1-(benzyloxy)-4-(chloromethyl)benzene (**19**) was synthesized according to the general procedure. Beige solid (50mg, 8%). R*_f_*  0.6 (CH_2_Cl_2_/AcOEt 1:1); mp: 79-81 ^o^C. ^1^H NMR (400 MHz, DMSO) δ 7.46 – 7.42 (m, 2H, Ar), 7.41 – 7.38 (m, 2H, Ar), 7.38 – 7.30 (m, 3H, Ar), 7.03 – 6.98 (m, 2H, Ar), 5.11 (s, 2H, OCH_2_), 4.72 (s, 2H, CH_2_Cl). ^13^C NMR (75 MHz, DMSO) δ 159 (C_4_), 136.7 (C_1’_), 130.1 (C_1_), 129.6 (C_2_, C_6_), 128.9 (C_3’_, C_5’_), 127.6 (C_4’_), 127.1 (C_2’_, C_6’_), 114.5 (C_3_, C_5_), 70.8 (OCH_2_), 46.2 (CH_2_Cl). Anal. Calcd for C_14_H_13_ClO: C, 72.26; H, 5.63. Found: C, 72.29; H, 5.65.

The compound 1-(tert-butyl)-4-((4-(chloromethyl)phenoxy)methyl)benzene (**20**) was synthesized according to the general procedure. Beige solid (305mg, 43.7%). R*_f_*  0.6 (CH_2_Cl_2_/AcOEt 1:1); mp: 79-81 ^o^C. ^1^H NMR (600 MHz, DMSO) δ 7.42 – 7.39 (m, 2H, Ar), 7.38 – 7.34 (m, 4H, Ar), 7.01 – 6.98 (m, 2H, Ar), 5.07 (s, 2H, OCH_2_), 4.71 (s, 2H, CH_2_Cl), 1.28 (s, 9H, t- Bu). ^13^C NMR (75 MHz, DMSO) δ 159 (C_4_), 150.2 (C_4’_), 133.6 (C_1’_), 130.1 (C_1_), 129.6 (C_2,_ C_6_), 126.7 (C_2’_, C_6’_), 125.2 (C_3’,_ C_5’_), 114.5 (C_3_, C_5_), 70.8 (OCH_2_), 46.2 (CH_2_Cl), 34.2 (C- t- Bu), 31.3 (t- Bu). Anal. Calcd for C_18_H_21_ClO: C, 74.86; H, 7.33. Found: C, 74.89; H, 7.39.

**Synthesis of *O*-Substituted Hydroxylamines**

General procedure:

To a solution of the appropriate N-hydroxyphthalimide (250.0 mg, 1 equiv.) in CH_2_Cl_2_ (3 mL), hydrazine monohydrate 64% w/w (2 equiv.) is added, and the reaction is stirred at rt for 1–24 h. The white precipitate formed is filtered, washed with CH_2_Cl_2_, and the filtrate is concentrated to afford the corresponding hydroxylamine.

The compound O-(4-(trifluoromethyl)benzyl)hydroxylamine (**21**) was synthesized from the compound **1** (2.52 g, 7.84 mmol, 1.0 eq) according to the general procedure (3 h), to afford after column chromatography purification a colorless oil (1.24 g, 83%). ^1^H NMR (400 MHz, CDCl_3_) δ 7.38-7.40 (m, 1H), 7.31-7.33 (m, 1H), 7.17-7.21 (m, 2H), 5.26 (brs, 2H), 4.76 (s, 2H) (3).

The compound O-(2,4-difluorobenzyl)hydroxylamine (**22**) was synthesized from the compound **2** (0,8643 mmol) according to the general procedure (3 h), to afford after column chromatography purification a colorless oil (104,8 mg, 76 %). ^1^H NMR (400 MHz, CDCl_3_) δ 6.97 (dd, *J* = 2.1, 1.1 Hz, 2H), 6.81 (t, *J* = 1.9 Hz, 1H), 5.07 (s, 2H), 4.64 (s, 2H), 2.24 (s, 6H). ^13^C NMR (125 MHz, CDCl_3_) δ 138.19, 133.91, 129.26, 126.23, 77.52, 21.07.

The compound O-(3,4-difluorobenzyl)hydroxylamine (**23**) was synthesized from the compound **3** according to the general procedure (3 h), ), to afford after column chromatography purification a colorless oil (109mg, 79 %). R*_f_* 0.12 (CH_2_Cl_2_). ^1^H NMR (400 MHz, CDCl_3_) δ 7.22 – 7.18 (m, 1H, Ar), 7.17 – 7.14 (m, 1H, Ar), 7.14 – 7.09 (m, 1H, Ar), 5.30 (s, 2H, NH_2_), 4.64 – 4.58 (m, 2H, OCH_2_). ^13^C NMR (101 MHz, CDCl_3_) *δ*: 149.0 (C_3_), 148.9 (C_4_), 129.9 (C_1_), 121.0 (C_6_), 111.6 (C_2_), 110.0 (C_5_), 77.8 (OCH_2_).

The compound O-(2,5-difluorobenzyl)hydroxylamine (**24**) was synthesized from the compound **4** according to the general procedure (3 h), to afford after column chromatography purification a colorless oil (159mg, 72 %). R*_f_* 0.14 (CH_2_Cl_2_). ^1^H NMR (400 MHz, DMSO) δ 7.30 – 7.11 (m, 3H, Ar), 6.22 (s, 2H, NH_2_), 4.61 (d, *J* = 1.4 Hz, 2H, OCH_2_). ^13^C NMR (126 MHz, CDCl_3_) δ 158.7 (C_5_), 155.2 (C_2_), 129.5 (C_1_), 117.3 (C_3_), 116 (C_4_), 115.7 (C_6_), 72.2 (OCH_2_).

The compound O-(4-(benzyloxy)benzyl)hydroxylamine (**25**) was synthesized from the compound **5** according to the general procedure (3 h), to afford after column chromatography purification a colorless oil (150mg, 99 %). R*_f_* 0.14 (CH_2_Cl_2_). ^1^H NMR (400 MHz, DMSO) δ 7.48 – 7.42 (m, 2H, Ar), 7.39 (ddd, *J* = 7.6, 6.7, 1.4 Hz, 2H, Ar), 7.35 – 7.29 (m, 1H, Ar), 7.27 – 7.20 (m, 2H, Ar), 7.02 – 6.92 (m, 2H, Ar), 5.94 (s, 2H, NH_2_), 5.09 (s, 2H, CH_2_), 4.47 (s, 2H, OCH_2_). ^13^C NMR (126 MHz, DMSO) δ 157.9 (C_4_), 136.7 (C_1’_), 129.3 (C_2_, C_6_), 128.9 (C_3’_, C_5’_), 128.8 (C_1_), 127.6 (C_4’_), 127.1 (C_2’_, C_6’_), 114.5 (C_3_, C_5_), 79.0 (OCH_2_), 70.8 (OCH_2_).

The compound O-(4-((4-(tert-butyl)benzyl)oxy)benzyl)hydroxylamine (**26**) was synthesized from the compound **6** according to the general procedure (3 h), to afford after column chromatography purification a colorless oil (135.10mg, 99%). R*_f_* 0.13 (CH_2_Cl_2_). ^1^H NMR (400 MHz, DMSO) δ 7.44 – 7.33 (m, 4H, Ar), 7.26 – 7.21 (m, 2H, Ar), 6.99 – 6.94 (m, 2H, Ar), 5.76 (s, 2H, NH_2_), 5.05 (s, 2H, OCH_2_), 4.48 (s, 2H, OCH_2_), 1.28 (d, *J* = 2.5 Hz, 9H, t-Bu). ^13^C NMR (126 MHz, DMSO) δ 157.9 (C_4_), 150.2 (C_4’_), 133.6 (C_1’_), 129.3 (C_2_, C_6_), 128.8 (C_1_), 126.7 (C_2’_,C_6’_), 125.2 (C_3’_, C_5’_), 114.5 (C_3,_ C_5_), 79.0 (OCH_2_), 70.8 (OCH_2_), 34.2 (C- (CH_3_)_3_), 31.3 (CH_3_).

The compound O-(4-(naphthalen-2-ylmethoxy)benzyl)hydroxylamine (**27**) was synthesized from the compound **7** according to the general procedure (3 h), to afford after column chromatography purification a colorless oil (99mg, 99%). R*_f_* 0.16 (CH_2_Cl_2_). ^1^H NMR (400 MHz, DMSO) δ 8.02 – 7.87 (m, 4H, Ar), 7.62 – 7.48 (m, 3H, Ar), 7.29 – 7.20 (m, 2H, Ar), 7.07 – 6.98 (m, 2H, Ar), 5.94 (s, 2H, NH_2_), 5.27 (s, 2H, OCH_2_), 4.48 (s, 2H, OCH_2_). ^13^C NMR (126 MHz, DMSO) δ 158.27 (C_4_), 135.11 (C_1’_), 133.14 (C_3’_), 132.89 (C_4’_), 130.71 (C_2_, C_6_), 130.42 (C_1_), 130.26 (C_6’_), 129.93 (C_10’_), 128.47 (C_7’_), 128.16 (C_5’_), 128.00 (C_2’_), 126.76 (C_8’_), 126.55 (C_9’_), 114.97 (C_3_, C_5_), 76.94 (OCH_2_), 69.64 (OCH_2_).

The compound O-(4-methylbenzyl)hydroxylamine (**28**) was synthesized from the compound **8** (0,94 mmol) according to the general procedure (3 h), to afford after column chromatography purification a colorless oil (128,3 mg, quantitative yield). ^1^H NMR (600 MHz, Chloroform-*d*) δ 7.27 (s, 1H), 7.25 (s, 1H), 7.18 (s, 1H), 7.17 (s, 1H), 4.66 (s, 2H), 2.36 (s, 3H) (4).

The compound O-(3-methylbenzyl)hydroxylamine (**29**) was synthesized from the compound **9** according to the general procedure (3 h), to afford after column chromatography purification a colorless oil (121.4 mg, 95 %). R*_f_* 0.14 (CH_2_Cl_2_).^1^H NMR (400 MHz, CDCl_3_) δ 7.20 – 7.10 (m, 4H, Ar), 4.67 (s, 2H, O CH_2_), 2.37 (d, *J* = 0.8 Hz, 3H, CH_3_).^13^C NMR (126 MHz, CDCl_3_) δ 161.17 (C_1_), 159.15 (C_3_), 132.96, 132.09 (C_2_, C_5_), 131.73 (C_4_), 120.68 (C_6_), 51.88 (OCH_2_), 21.77 (CH_3_).

The compound O-(2-methylbenzyl)hydroxylamine (**30**) was synthesized from the compound **10** (0,8643 mmol) according to the general procedure (3 h), to afford after column chromatography purification a colorless oil (169.7 mg, 95 %). R*_f_* 0.12 (CH_2_Cl_2_). ^1^H NMR (600 MHz, CDCl_3_) δ 7.34 – 7.28 (m, 1H, Ar), 7.25 – 7.16 (m, 3H, Ar), 5.40 (s, 2H, NH_2_), 4.73 (s, 2H, OCH_2_), 2.38 (s, 3H, CH_3_). ^13^C NMR (125 MHz, CDCl_3_) δ 136.71 (C_1_), 135.27 (C_2_), 129.67 (C_6_), 129.34, 128.42 (C_3_, C_4_), 126.59 (C_5_), 76.76 (OCH_2_), 19.24 (CH_3_).

The compound O-(naphthalen-2-ylmethyl)hydroxylamine (**31**) was synthesized from the compound **11** according to the general procedure (3 h), to afford after column chromatography purification a colorless oil (224mg, 98%). R*_f_* 0.14 (CH_2_Cl_2_). ^1^H NMR (400 MHz, DMSO) δ 7.95 – 7.79 (m, 4H, Ar), 7.57 – 7.40 (m, 3H, Ar), 6.11 (s, 2H, NH_2_), 4.74 (s, 2H, OCH_2_). ^13^C NMR (126 MHz, CDCl_3_) δ 134.9 (C_1_), 133.3 (C_3_), 133.2 (C_4_), 128.3 (C_6_), 128.0 (C_10_), 127.7 (C_7_), 127.4 (C_5_), 126.2 (C_2_), 126.1 (C_8_), 125.1 (C_9_), 78.1 (OCH_2_).

The compound O-(3-fluorobenzyl)hydroxylamine (**32**) was synthesized from the compound **12** according to the general procedure (3 h), to afford after column chromatography purification a colorless oil (159mg, 72 %). R*_f_* 0.14 (CH_2_Cl_2_). ^1^H NMR (400 MHz, DMSO) δ 7.30 – 7.11 (m, 3H, Ar), 6.22 (s, 2H, NH_2_), 4.61 (d, *J* = 1.4 Hz, 2H, OCH_2_). ^13^C NMR (126 MHz, CDCl_3_) δ 158.7 (C_5_), 155.2 (C_2_), 129.5 (C_1_), 117.3 (C_3_), 116 (C_4_), 115.7 (C_6_), 72.2 (OCH_2_).

The compound O-(3-bromobenzyl)hydroxylamine (**33**) was synthesized from the compound **14** according to the general procedure (3 h), to afford after column chromatography purification a colorless oil (145.6 mg, 96 %). R*_f_* 0.30 (CH_2_Cl_2_). ^1^H NMR (600 MHz, CDCl_3_) δ 7.53 (t, *J* = 1.9 Hz, 1H, Ar), 7.44 (ddd, *J* = 7.8, 1.9, 1.1 Hz, 1H, Ar), 7.31 – 7.26 (m, 1H, Ar), 7.23 (t, *J* = 7.7 Hz, 1H, Ar), 5.45 (s, 2H, NH_2_), 4.65 (s, 2H, OCH_2_). ^13^C NMR (100 MHz, CD_3_OD) δ 136.78 (C_1_), 133.62 (C_2_), 133.14 (C_4_), 131.71 (C_5_), 129.02 (C_6_), 123.59 (C_3_), 77.03 (OCH_2_).

The compound O-(4-bromobenzyl)hydroxylamine (**34**) was synthesized from the compound **6** according to the general procedure (3 h), to afford after column chromatography purification a colorless oil (180 mg, 99 %). R*_f_* 0.13 (CH_2_Cl_2_). ^1^H NMR (400 MHz, CDCl_3_) δ 7.55 – 7.49 (m, 2H, Ar), 7.31 – 7.27 (m, 2H, Ar), 5.52 – 5.38 (m, 2H, NH_2_), 4.67 (s, 2H, OCH_2_). ^13^C NMR (100 MHz, CDCl_3_) *δ*: 136.6 (C_1_), 131.5 (C_5_, C_3_), 130.0 (C_6_, C_2_), 121.8 (C_4_), 77.0 (OCH_2_).

The compound O-(2-bromobenzyl)hydroxylamine (**35**) was synthesized from the compound **15** according to the general procedure (3 h), to afford after column chromatography purification a colorless oil (183 mg, 100 %). R*_f_* 0.12 (CH_2_Cl_2_). ^1^H NMR (400 MHz, CDCl_3_) δ 7.59 – 7.48 (m, 1H), 7.44 (dd, *J* = 7.7, 1.8 Hz, 1H), 7.32 (td, *J* = 7.5, 1.3 Hz, 1H), 7.17 (td, *J* = 7.7, 1.9 Hz, 1H), 5.61 – 5.39 (m, 2H), 4.81 (s, 2H). ^13^C NMR (75 MHz, CDCl_3_) d 136.90 (C_1_), 132.74, 129.95, 129.31 (C_4_, C_3_, C_6_), 127.35 (C_5_), 123.54 (C_2_), 77.14 (OCH_2_).

The compound O-(3-chlorobenzyl)hydroxylamine (**36**) was synthesized from the compound **16** according to the general procedure (3 h), to afford after column chromatography purification a colorless oil (190.5 mg, 99 %). R*_f_* 0.27 (CH_2_Cl_2_). ^1^H NMR (400 MHz, CDCl_3_) δ 7.37 (q, *J* = 1.3 Hz, 1H, Ar), 7.29 (dd, *J* = 5.3, 1.2 Hz, 2H, Ar), 7.25 – 7.21 (m, 1H, Ar), 5.45 (s, 2H, NH_2_), 4.66 (s, 2H, OCH_2_). ^13^C NMR (100 MHz, CDCl_3_) *δ*: 139.8 (C_1_), 134.3 (C_3_), 129.7 (C_5_), 128.2 (C_4_), 128.0 (C_2_), 126.2 (C_6_), 76.9 (OCH_2_).

The compound O-(3,4-dimethoxybenzyl)hydroxylamine (**37**) was synthesized from the compound **17** according to the general procedure (3 h), to afford after column chromatography purification a colorless oil (144 mg, 99 %). R*_f_* 0.10 (CH_2_Cl_2_). ^1^H NMR (400 MHz, CDCl_3_) δ 6.94 – 6.89 (m, 2H, Ar), 6.85 (d, *J* = 8.6 Hz, 1H, Ar), 5.36 (s, 2H, NH_2_), 4.63 (d, *J* = 0.6 Hz, 2H, OCH_2_), 3.89 (d, *J* = 5.6 Hz, 6H, CH_3_).  ^13^C NMR (100 MHz, CDCl_3_) *δ*: 149.0 (C_3_), 148.9 (C_4_), 129.9 (C_1_), 121.0 (C_6_), 111.6 (C_2_), 110.0 (C_5_), 77.8 (OCH_2_), 55.8 (OCH_3_).

**Synthesis of *N*-Hydroxypyridinediones 1235, 1464, 1617, 1810, 1738, 1808, 1899, 1462, 1620, 1681, 1811, 1466, 1618, 1621, 1717, 1718, 1719 and 1669**

General procedure:

To a solution of the appropriate hydroxylamine (0.57 mmol, 1.05 equiv.) in abs. EtOH (2 mL), 5-acetyl-1,6-dihydroxy-4-methylpyridin-2(1*H*)-one (B) (0.55 mmol, 1 equiv.) is added and the reaction mixture is stirred at RT, under argon, overnight. Thereafter, the solvent is evaporated under vacuum. The solid residue is triturated with Et_2_O or Et_2_O/AcOEt (9/1) under ice to afford the desired compound as a solid.

The compound 1,6-dihydroxy-4-methyl-5-(1-(((4-(trifluoromethyl)benzyl)oxy)imino)ethyl)pyri-din-2(1*H*)-one (**1235**) was synthesized from the compound **21** (329 mg, 1.72 mmol, 1.05 eq) according to the general procedure. Yellow solid (135 mg, 23%). mp 99-101 °C (AcOEt/dry Et_2_O), R*_f_* = 0.10 (AcOEt), R*_f_* _(_*_RP-TLC_*_)_ = 0.03 (H_2_O/ACN 7:3); ^1^H NMR (600.11 MHz, DMSO-*d_6_*) *δ* (ppm) 1.73, 1.77 (s + s, 0.6H, 4-C*H_3_*), 1.86, 1.89 (s + s, 2.2H, 4-C*H_3_*), 1.94, 1.97 (s + s, 0.9H, 7-C*H_3_*), 2.01, 2.05 (s + s, 1.9H, 7-C*H_3_*), 5.06, 5.10 (s +s, 0.6H, OC*H_2_*4-CF_3_C_6_H_4_), 5.19, 5.22 (s +s, 1.4H, OC*H_2_*4-CF_3_C_6_H_4_), 5.48, 5.53 (s + s, 1H, H_3_), 7.50 (d, 0.6H, *J*=7.9 Hz, H_2'_, H_6'_), 7.57 (d, 1.4H, *J*=8.0 Hz, H_2'_, H_6'_), 7.68 (d, 0.6H, *J*=7.8 Hz, H_3'_, H_5'_), 7.71 (d, 1.4H, *J*=8.0 Hz, H_3'_, H_5'_), 10.19 (low), 11.47 (s + v br s, 1H, 1-OH, 6-OH); ^13^C NMR (150.9 MHz, DMSO-*d_6_*) *δ* (ppm) 15.9 (7-*C*H_3_), 19.3, 19.4 (4-*C*H_3_), 19.8 (7-*C*H_3_), 73.5, 73.6, 73.9 (O*C*H_2_4-CF_3_C_6_H_4_), 90.6, 91.0 (C_3_), 110.3, 112.0 (C_5_), 123.3, 125.1 (d, *J_C-F_*=269.5 Hz, *C*F_3_), 125.00, 125.03 (d, *J_C-F_*=3.8 Hz, C_3'_, C_5'_), 127.9, 128.06 (d, *J_C-F_*=30.0 Hz, C_4'_), 128.06, 128.11 (C_2'_, C_6'_), 143.3, 143.7 (C_1'_), 145.6, 146.9 (C_4_), 153.1, 153.7 (C_7_), 154.4, 154.9 (C_6_), 156.4 (C_2_). Anal. Calcd for C_16_H_15_F_3_N_2_O_4_: C, 53.94; H, 4.24; N, 7.86. Found: C, 54.00; H, 4.28; N, 7.88.

The compound 5-(1-(((2,4-difluorobenzyl)oxy)imino)ethyl)-1,6-dihydroxy-4-methylpyridin-2(1*H*)-one (**1464**) was synthesized from the compound **22** (96.0 mg, 0,60 mmol ) according to the general procedure. Green solid (84,8 mg, 48 %). mp 102-104 ^o^C (dec.), R*_f_* = 0.05 (EtOAc/MeOH 3:1). ^1^H NMR (600 MHz, DMSO) δ 7.53 – 7.43 (m, 1H), 7.25 – 7.18 (m, 1H), 7.10 – 7.04 (m, 1H), 5.49 (d, *J* = 11.1 Hz, 1H), 5.12 (d, *J* = 21.8 Hz, 2H), 1.97 (d, *J* = 13.3 Hz, 3H), 1.85 (d, *J* = 14.6 Hz, 3H). ^13^C NMR (151 MHz, DMSO) δ 156.50 (C_2_), 154.07 (C_6_), 151.87 (C_7_), 146.63 (C_2‘_), 133.86 (C_4‘_), 133.94 (C_5‘_), 131.88 (C_3‘_), 131.03 (C_1‘_), 130.90 (C_6‘_), 127.34 (C_3_), 127.00 (C_5_), 92.08 (C_4_), 61.31 (-CH_2_-), 22.53 (4-CH_3_), 17.91 (7-CH_3_) Anal. Calcd for C_15_H_14_F_2_N_2_O_4_: C, 55.56; H, 4.35; N, 8.64. Found: C, 55.58; H, 4.32; N, 8.67.

The compound 5-(1-(((3,4-difluorobenzyl)oxy)imino)ethyl)-1,6-dihydroxy-4-methylpyridin-2(1*H*)-one (**1617**) was synthesized from the compound **23** according to the general procedure. light green solid (122.4 mg, 70 %). (50.0mg, 31.4%). R*_f_* 0.10 (ΑcOEt). mp: 120-122 ^o^C dec. ^1^H NMR (600 MHz, DMSO) δ 7.46 – 7.29 (m, 2H, Ar), 7.24 – 7.09 (m, 1H, Ar), 5.46 (s, *J* = 32.7 Hz, 1H, H_3_), 5.01 (s, *J* = 72.2 Hz, 2H, OCH_2_), 1.99 (s, *J* = 33.0 Hz, 3H, CH_3_), 1.87 (s, 3H, CH_3_). ^13^C NMR (151 MHz, DMSO) δ 156.5 (C_2_), 154.37 (C_6_), 153.64 (C_7_), 148.7 (C_3’_), 147.4 (C_4’_), 147.00 (C_4_), 137.4 (C_1_’), 124.82 (C_6’_), 117.67 (C_5’_), 116.92 (C_2’_), 111.54 (C_5_), 91.63 (C_3_), 73.47 (OCH_2_Ph), 20.30, (7- CH_3_), 19.87 (4- CH_3_), 16.36 (7- CH_3_). Anal. Calcd for C_15_H_14_F_2_N_2_O_4_: C, 55.56; H, 4.35; N, 8.64. Found: C, 55.60; H, 4.36; N, 8.67.

The compound 5-(1-(((2,5-difluorobenzyl)oxy)imino)ethyl)-1,6-dihydroxy-4-methylpyridin-2(1*H*)-one (**1810**) was synthesized from the compound **24** according to the general procedure. Blue solid (212mg, 76.3%). R*_f_* 0.10 (ΑcOEt). mp: 105-107 ^o^C. ^1^H NMR (400 MHz, DMSO) δ 7.35 – 7.07 (m, 3H, Ar), 5.5 (s, 1H, H_3_), 5.22 – 4.97 (s, 2H, OCH_2_), 2.38 – 2.24 (s, 1H, CH_3_), 2.07 – 1.92 (s, 3H, CH_3_), 1.87 – 1.69 (s, 2H, CH_3_). ^13^C NMR (126 MHz, DMSO) δ 164.03 (C_5’_), 159.24 (C_2’_), 157.26 (C_2_), 155.91 (C_6_) 154.03 (C_7_), 148.51 (C_4_), 145.84 (C_1’_), 139.90 (C_3’_), 128.50 (C_4’_), 117.01 (C_6’_), 114.75, 108.90, 108.34 (C_5_), 102.65, 98.36 (C_3_), 68.16 (OCH_2_), 25.44 (7- CH_3_), 20.28 (4- CH_3_), 16.76 (7- CH_3_). Anal. Calcd for C_15_H_14_F_2_N_2_O_4_: C, 55.56; H, 4.35; N, 8.64. Found: C, 55.58; H, 4.37; N, 8.67.

The compound 5-(1-(((4-(benzyloxy)benzyl)oxy)imino)ethyl)-1,6-dihydroxy-4-methylpyridin-2(1*H*)-one (**1738**) was synthesized from the compound **25** according to the general procedure. Green solid (80mg, 52 %). R*_f_* 0.10 (ΑcOEt). mp: 110-112 ^o^C. ^1^H NMR (600 MHz, DMSO) δ 7.52 – 7.33 (m, 5H, Ar), 7.33 – 7.12 (m, 3H, Ar), 6.97 (dtd, *J* = 13.0, 6.6, 2.8 Hz, 1H, Ar), 5.5 (s, 1H, H_3_), 5.17 – 5.06 (s, 2H, OCH_2_), 5.04 – 4.83 (s, 2H, OCH_2_), 2.41 – 2.24 (s, 3H, CH_3_), 2.03 – 1.84 (s, 3H, CH_3_). ^13^C NMR (126 MHz, DMSO) δ 156.5 (C_2_), 154.4 (C_6_), 153.8 (C_7_), 146.8 (C_4_), 146.37 (C_4’_), 138.6 (C_1’_), 133.5 (C_1’’_), 128.63 (C_2’_, C_6’_), 127.8 (C_3’’_, C_5’’_), 126.5 (C_4’’_), 126.0 (C_2’’_, C_6’’_), 115.12 (C_3’_, C_5’_), 114.69 (C_5_), 92.05 (C_3_), 69.31 (OCH_2_), 64.5 (OCH_2_), 25.19 (7- CΗ_3_), 19.5 (4- CH_3_). Anal. Calcd for C_22_H_22_N_2_O_5_: C, 66.99; H, 5.62; N, 7.10. Found: C, 67.00; H, 5.64; N, 7.12.

The compound 5-(1-(((4-((4-(tert-butyl)benzyl)oxy)benzyl)oxy)imino)ethyl)-1,6-dihydroxy-4-methylpyridin-2(1*H*)-one (**1808**) was synthesized from the compound **26** according to the general procedure. Green solid (143.2mg, 74 %). R*_f_* 0.10 (ΑcOEt). mp: 95-97 ^o^C. ^1^H NMR (600 MHz, DMSO) δ 7.45 – 7.33 (m, 4H, Ar), 7.32 – 7.25 (m, 1H, Ar), 7.18 (dd, *J* = 50.4, 8.1 Hz, 1H, Ar), 6.96 (dq, *J* = 10.8, 7.4 Hz, 2H, Ar), 5.5 (s, 1H, H_3_), 5.13 – 5.02 (s, 2H, OCH_2_), 5.01 – 4.82 (s, 2H, OCH_2_), 2.45 – 2.12 (s, 1H, CH_3_), 2.04 – 1.86 (s, 2H, CH_3_), 1.83 – 1.62 (s, 3H, CH_3_), 1.29 (s, *J* = 2.3 Hz, 9H, *t*-Bu). ^13^C NMR (126 MHz, DMSO) δ 157.78 (C_2_), 155.95 (C_6_), 150.79 (C_7_), 143.80 (C_4_), 134.82 (C_4’_), 133.89 (C_1’’_), 129.98 (C_1’_), 129.72 (C_2’_, C_6’_), 128.19 (C_4’’_), 128.03 (C_2’’_, C_6’’_), 127.82 (C_3’’_, C_5’’_), 125.43, 120.16 (C_3’_, C_5’_), 115.27 (C_5_), 106.55 (C_3_), 66.86 (OCH_2_), 62.78 (OCH_2_), 34.57 (C- t-Bu), 31.37 (t-Bu), 25.13 (7- CH_3_), 21.15 (4- CH_3_). Anal. Calcd for C_26_H_30_N_2_O_5_: C, 69.31; H, 6.71; N, 6.22. Found: C, 69.33; H, 6.74; N, 6.24.

The compound 1,6-dihydroxy-4-methyl-5-(1-(((4-(naphthalen-2-ylmethoxy)benzyl)oxy)imi-no)ethyl)pyridin-2(1*H*)-one (**1899**) was synthesized from the compound **27** according to the general procedure. Green solid (220mg, 65 %). R*_f_* 0.10 (ΑcOEt). mp: 95-97 ^o^C. ^1^H NMR (400 MHz, DMSO) δ 7.94 (dd, *J* = 17.4, 8.1 Hz, 4H, Ar), 7.60 – 7.48 (m, 3H, Ar), 7.34 – 7.16 (m, 2H, Ar), 7.10 – 6.92 (m, 2H, Ar), 5.43 (s, 1H, H_3_), 5.27 (s, *J* = 7.1 Hz, 2H, OCH_2_), 5.11 – 4.84 (s, 2H, OCH_2_), 2.31 (s, 3H, CH_3_), 1.99 – 1.82 (s, 3H, CH_3_). ^13^C NMR (101 MHz, DMSO) δ 165.36 (C_2_), 159.40 (C_6_), 158.41 (C_7_), 149.67 (C_4_), 135.48 (C_4’_), 133.40 (C_1’’_), 133.15 (C_5’’_), 128.67 (C_4’’_), 128.55 (C_1’_), 128.38 (C_2’_, C_6’_), 128.22 (C_2’’_), 126.93 (C_3_), 126.73 (C_7’’_), 126.26 (C_10’’_), 120.19 (C_3’’_), 115.28 (C_6’’_), 107.65 (C_9’’_), 105.29 (C_8’’_), 100.54 (C_3’_, C_5’_), 95.3 (C_5_), 74.85 (OCH_2_), 69.92 (OCH_2_), 20.14 (7- CH_3_), 15.78 (4- CH_3_). Anal. Calcd for C_26_H_24_N_2_O_5_: C, 70.26; H, 5.44; N, 6.30. Found: C, 70.27; H, 5.48; N, 6.32.

The compound 1,6-dihydroxy-4-methyl-5-(1-(((4-methylbenzyl)oxy)imino)ethyl)pyridin-2(1*H*)-one (**1462**) was synthesized from the compound **28** (78,6 mg, 0,5733 mmol, 1.05 eq) according to the general procedure. Green solid (73,1 mg, 44 %). mp 138-140 ^o^C, R*_f_* = 0.07 (EtOAc/MeOH 3:1). ^1^H NMR (600 MHz, DMSO) δ 7.27 – 7.23 (m, 2H, Ar), 7.21 – 7.18 (m, 2H, Ar), 5.36 (s, 1H, H3), 4.73 (s, 2H, -CH_2_-), 2.45 (s, 3H, 7-CH_3_), 2.31 (s, 3H, 4-CH_3_), 2.24 (s, 3H, 4’-CH_3_). ^13^C NMR (151 MHz, DMSO) δ 159.40 (C_2_), 153.67 (C_6_), 150.45 (C_7_), 128.90 (C_3’,_ C_5’_), 128.69 (C_1’_), 128.62 (C_4’_), 127.79 (C_2’_, C_6’_), 91.08 (C_3_), 78.37 (C_4_), 76.29 (C_5_), 74.47 (-CH_2_-), 20.69 (7-CH_3_), 19.52 (4-CH_3_), 15.83 (4’-CH_3_). Anal. Calcd for C_16_H_18_N_2_O_4_: C, 63.56; H, 6.00; N, 9.27; Found: C, 63.58; H, 6.02; N, 9.25.

The compound 1,6-dihydroxy-4-methyl-5-(1-(((3-methylbenzyl)oxy)imino)ethyl)pyridin-2(1*H*)-one (**1620**) was synthesized from the compound **29** according to the general procedure. Green solid (59.8 mg, 42 %). R*_f_* 0.10 (ΑcOEt). mp: 140-142 ^o^C (dec.). ^1^H NMR (600 MHz, DMSO) δ 7.25 – 7.09 (m, 4H, Ar), 5.50 (d, *J* = 14.8 Hz, 1H, CHC=ON), 5.05 (s, 2H, OCH_2_), 2.30 (s, 3H, PhCH_3_), 1.99 (d, *J* = 23.3 Hz, 3H, CH_3_C=N), 1.89 (d, *J* = 16.8 Hz, 3H, CH_3_). ^13^C NMR (126 MHz, DMSO) δ 156.54 (C_2_), 154.58 (C_6_), 154.17 (C_6_), 153.74, 153.70, 153.57 (C_7_), 146.98, 145.75 (C_4_), 138.51, 138.13, 137.32, 137.28(C_1’_), 128.59, 128.42 (C_2’_), 128.40, 128.27 (C_3’_), 128.25, 128.21 (C_5’_), 128.16, 128.14, 128.12(C_4’_), 125.03, 124.88(C_6’_), 112.37, 110.47 (C_5_), 90.99, 90.63 (C_3_), 79.27, 79.01, 78.74, 74.64, 74.62(OCH_2_Ph), 21.02, 21.00 (7-CH_3_), 19.90, 19.57 (7-CH_3_), 19.43 (7-CH_3_), 15.98 (3’-CH_3_). Anal. Calcd for C_16_H_18_N_2_O_4_: C, 63.56; H, 6.00; N, 9.27. Found: C, 63.59; H, 6.04; N, 9.31.

The compound 1,6-dihydroxy-4-methyl-5-(1-(((2-methylbenzyl)oxy)imino)ethyl)pyridin-2(1*H*)-one (**1681**) was synthesized from the compound **30** according to the general procedure. Light blue solid (79.4 mg, 25 %). R*_f_* 0.25 (ΑcOEt). mp: 108-110 ^o^C. ^1^H NMR (600 MHz, DMSO) δ 7.31 (dd, *J* = 14.6, 7.2 Hz, 1H, Ar), 7.21 – 7.15 (m, 3H, Ar), 5.20 – 4.86 (m, 3H. CHC=ON, OCH_2_), 2.34 – 2.20 (m, 6H, PhCH_3_, CH_3_C=N), 2.02 – 1.98 (m, 3H, CH_3_). ^13^C NMR (151 MHz, DMSO) δ 129.23 (C_1’_), 129.93 (C_3’_), 128.99 (C_6’_), 126.65 (C_4’_, C_5’_), 73.66 (C_3_), 73.42, 73.42 (OCH_2_Ph), 19.02 (7-CH_3_), 19.02 (4-CH_3_), 16.44 (2’-CH_3_). Anal. Calcd for C_16_H_18_N_2_O_4_: C, 63.56; H, 6.00; N, 9.27. Found: C, 63.60; H, 6.04; N, 9.30.

The compound 1,6-dihydroxy-4-methyl-5-(1-((naphthalen-2-ylmethoxy)imino)ethyl)pyridin-2(1*H*)-one (**1811**) was synthesized from the compound **31** according to the general procedure. Light blue solid (80mg, 51.5%). R*_f_* 0.10 (ΑcOEt). mp: 116-118 ^o^C. ^1^H NMR (600 MHz, DMSO) δ 7.96 – 7.77 (m, 4H, Ar), 7.61 – 7.37 (m, 3H, Ar), 5.5 (s, 1H, H_3_), 5.37 – 5.09 (s, 2H, OCH_2_), 2.41 – 2.25 (s, 1H, CH_3_), 2.14 – 2.02 (s, 2H, CH_3_), 2.01 – 1.92 (s, 1H, CH_3_), 1.92 – 1.72 (s, 2H, CH_3_). ^13^C NMR (126 MHz, DMSO) δ 166.00, 159.35 (C_2_), 153.62 (C_6_) ,152.71 (C_7_), 143.4 (C_4_), 135.87 (C_1’_) , 133.06 (C_5’_), 132.71 (C_4’_), 128.02 (C_2’_), 127.86 (C_7’_), 127.82 (C_10’_), 126.76 (C_3’_), 126.43 (C_6’_), 126.20 (C_9’_), 116.55 (C_8’_), 109.75 (C_5_), 98.2 (C_3_), 75.68 (OCH_2_), 25.19 (7- CH_3_), 19.5 (4- CH_3_). Anal. Calcd for C_19_H_18_N_2_O_4_: C, 67.45; H, 5.36; N, 8.28. Found: C, 67.46; H, 5.37; N, 8.30.

The compound 1,6-hydroxy-4-methyl-5-(1-(((4'-chlorobenzyl)oxy)imino)ethyl)pyridin-2(1H)-one (**1466**) was synthesized from the compound **7** (94.60 mg, 0.60 mmol) according to the general procedure. Yellow solid (122.4 mg, 69.5 %). R*_f_* = 0.10 (EtOAc/MeOH 3:1), mp 110 °C. ^1^H NMR (600 MHz, DMSO) δ 7.43 – 7.29 (m, 4H, Ar), 5.50 (s, J = 16.7 Hz, 1H, H_3_), 5.02 (s, J = 76.7 Hz, 2H, OCH_2_), 2.04 – 1.92 (s, 3H, CH_3_), 1.87 (s, J = 9.5 Hz, CH_3_). ^13^C NMR (151 MHz, DMSO) δ 156.53 (C_2_), 154.6, 154.57 (C_6_), 153.79 (C_7_), 146.8 (C_4_), 138.6 (C_1’_), 132.18 (C_4’_), 129.63 (C_2’_, C_6’_), 128.27 (C_3’_, C_5’_), 112.23 (C_5_), 91.13 (C_3_), 90.70, 73.76 (OCH_2_Ph), 23.43, 19.95 (7- CH_3_), 19.60 (4- CH_3_), 15.99 (7- CH_3_). Anal. calcd (%) for C_15_H_15_ClN_2_O_4_: C, 55.82; H, 4.68; N, 8.68. Found: C, 55.83; H, 4.70; N, 8.69.

The compound 5-(1-(((3-fluorobenzyl)oxy)imino)ethyl)-1,6-dihydroxy-4-methylpyridin-2(1*H*)-one (**1618**) was synthesized from the compound **32** according to the general procedure. Green solid (61.8mg, 41.06%). R*_f_* 0.10 (ΑcOEt). mp: 110-112 ^o^C. ^1^H NMR (600 MHz, DMSO) δ 7.42 – 7.34 (m, 1H, Ar), 7.22 – 7.06 (m, 3H, Ar), 5.49 (s, *J* = 28.9 Hz, 1H, H_3_), 5.04 (s, *J* = 75.1 Hz, 2H, OCH_2_), 2.00 (s, *J* = 37.3 Hz, 3H, CH_3_), 1.88 (s, 3H, CH_3_). ^13^C NMR (151 MHz, DMSO) δ 163.20 (C_3’_), 161.59, 156.71 (C_2_), 155.02 (C_6_), 154.07 (C_7_), 147.05 (C_4_), 145.80 (C_1’_), 142.09, 130.45 (C_5’_), 123.70 (C_6’_), 114.41 (C_4’_), 114.27 (C_2’_), 112.03 (C_5_), 91.36 (C_3_), 90.87, 73.89 (OCH_2_Ph), 20.13 (7- CH_3_), 19.72 (4- CH_3_), 16.19 (7- CH_3_). Anal. Calcd for C_15_H_15_FN_2_O_4_: C, 58.82; H, 4.94; N, 9.15. Found: C, 58.84; H, 4.95; N, 9.17.

The compound 5-(1-(((3-bromobenzyl)oxy)imino)ethyl)-1,6-dihydroxy-4-methylpyridin-2(1*H*)-one (**1621**) was synthesized from the compound **33** according to the general procedure. Green solid (87.4 mg, 49 %). R*_f_* 0.30 (ΑcOEt). mp: 120-122 ^o^C (dec.). ^1^H NMR (400 MHz, DMSO) δ 7.54 – 7.45 (m, 2H, Αr), 7.38 – 7.29 (m, 2H, Ar), 5.45 (d, *J* = 24.4 Hz, 1H, CHC=ON), 5.02 (d, *J* = 51.5 Hz, 2H, OCH_2_), 1.99 (d, *J* = 23.3 Hz, 3H, CH_3_C=N), 1.89 – 1.73 (m, 3H, CH_3_). ^13^C NMR (151 MHz, DMSO) δ 156.47 (C_3’_), 154.54 (C_2_), 153.94 (C_7_), 146.74 (C_4_), 141.67, 141.60 (C_1’_), 130.39 (C_2’_), 130.20 (C_6’_), 126.53 (C_4’_), 121.47 (C_5’_), 91.21 (C_3_), 73.45 (OCH_2_), 19.89 ((7-CH_3_), 19.50 (4-CH_3_), 15.93 (7-CH_3_). Anal. Calcd for C_15_H_15_BrN_2_O_4_: C, 49.06; H, 4.12; N, 7.63. Found: C, 49.10; H, 4.16; N, 7.67.

The compound 5-(1-(((4-bromobenzyl)oxy)imino)ethyl)-1,6-dihydroxy-4-methylpyridin-2(1*H*)-one (**1717**) was synthesized from the compound **34** according to the general procedure. Light blue solid (93.6 mg, 33 %). R*_f_* 0.25 (ΑcOEt). mp: 142-144 ^o^C (dec.). ^1^H NMR (600 MHz, DMSO) δ 7.54 (q, *J* = 7.4, 6.7 Hz, 2H, Αr), 7.36 – 7.24 (m, 2H, Ar), 5.58 – 5.22 (m, 1H, CHC=ON), 5.08 – 4.90 (m, 2H, OCH_2_), 2.35 – 2.27 (m, 1H, CH_3_C=N), 2.04 – 1.95 (m, 3H, CH_3_), 1.79 (d, *J* = 33.4 Hz, 2H, CH_3_C=N). ^13^C NMR (151 MHz, DMSO) δ 131.03 (C_1’_), 129.96 (C_2’_, C_6’_), 129.79, 128.52 (C_3’_, C_5’_), 73.49 (C_3_), 64.85 (OCH_2_), 19.49 (7-CH_3_), 15.11 (4-CH_3_). Anal. Calcd for C_15_H_15_BrN_2_O_4_: C, 49.06; H, 4.12; N, 7.63. Found: C, 49.09; H, 4.15; N, 7.66.

The compound 5-(1-(((2-bromobenzyl)oxy)imino)ethyl)-1,6-dihydroxy-4-methylpyridin-2(1*H*)-one (**1718**) was synthesized from the compound **35** according to the general procedure. Light blue solid (95.5 mg, 34 %). R*_f_* 0.25 (ΑcOEt). mp: 148-150 ^o^C (dec.). ^1^H NMR (600 MHz, DMSO) δ 7.67 – 7.57 (m, 1H, Αr), 7.47 – 7.37 (m, 2H, Ar), 7.24 (ddt, *J* = 25.0, 17.5, 7.4 Hz, 1H, Ar), 5.61 – 5.38 (m, 1H, CHC=ON), 5.23 – 4.95 (m, 2H, OCH_2_), 2.33 – 2.28 (m, 1H, CH_3_C=N), 2.10 – 1.96 (m, 3H, CH_3_), 1.88 – 1.75 (m, 1H, CH_3_C=N). ^13^C NMR (151 MHz, DMSO) δ 153.75 (C_2_), 146.77 (C_4_), 132.35 (C_1’_), 131.91 (C_3’_), 129.91 (C_6’_), 129.38, 128.59 (C_4’_), 128.18, 127.57 (C_5’_), 73.70 (C_3_), 62.60 (OCH_2_), 25.19 (7-CH_3_), 16.86 (4-CH_3_). Anal. Calcd for C_15_H_15_BrN_2_O_4_: C, 49.06; H, 4.12; N, 7.63. Found: C, 49.10; H, 4.16; N, 7.66.

The compound 1,6-dihydroxy-4-methyl-5-(1-(((3-chlorobenzyl)oxy)imino)ethyl)pyridin-2(1*H*)-one (**1719**) was synthesized from the compound **36** according to the general procedure. Light blue solid (156.6 mg, 47 %). R*_f_* 0.25 (ΑcOEt). mp: 105-107 ^o^C. ^1^H NMR (600 MHz, DMSO) δ 7.44 – 7.26 (m, 4H, Αr), 5.58 – 5.17 (m, 1H, CHC=ON), 5.16 – 4.93 (m, 2H, OCH_2_), 2.34 – 2.27 (m, 1H, CH_3_C=N), 2.06 – 1.94 (m, 3H, CH_3_), 1.86 – 1.74 (m, 2H, CH_3_C=N). ^13^C NMR (151 MHz, DMSO) δ 146.72 (C_7_), 145.60 (C_4_), 132.88 (C_3’_), 130.06 (C_1’_), 127.27 (C_2’_), 126.10 (C_4’_, C_5’_, C_6’_), 92.05 (C_3_), 73.38 (OCH_2_), 19.69 (7-CH_3_), 16.11 (4-CH_3_). Anal. Calcd for C_15_H_15_ClN_2_O_4_: C, 55.82; H, 4.68; N, 8.68. Found: C, 55.86; H, 4.72; N, 8.72.

The compound 5-(1-(((3,4-dimethoxybenzyl)oxy)imino)ethyl)-1,6-dihydroxy-4-methylpyridin-2(1*H*)-one (**1669**) was synthesized from the compound **37** according to the general procedure. Light green solid (199.7 mg, 81 %). R*_f_* 0.20 (ΑcOEt). mp: 120-122 ^o^C (dec.). ^1^H NMR (600 MHz, DMSO) δ 6.97 – 6.82 (m, 3H, Ar), 5.44 (d, *J* = 21.7 Hz, 1H, CHC=ON), 5.02 – 4.87 (m, 2H, OCH_2_), 3.75 – 3.72 (m, 6H, 2 x OCH_3_), 1.98 (d, *J* = 14.3 Hz, 3H, CH_3_C=N), 1.89 (d, *J* = 27.3 Hz, 3H, CH_3_). ^13^C NMR (126 MHz, DMSO) δ 156.95(C_3’_, C_4’_), 154.97 (C_2_), 154.46 (C_6_), 148.94, 148.93, 148.91 (C_7_), 148.86, 148.68, 148.66 (C_4_), 131.27, 130.89 (C_1’_), 130.51 (C_2’_), 120.75 (C_6’_), 112.13 (C_5’_), 111.91, 111.88 (C_5_), 91.41, 91.05 (C_3_), 75.02, 74.90 (OCH_2_), 31.12 (OCH_3_), 20.27 (7-CH_3_), 20.01, 19.84 (4-CH_3_), 16.39 (7-CH_3_). Anal. Calcd for C_17_H_20_N_2_O_6_: C, 58.61; H, 5.79; N, 8.04. Found: C, 58.65; H, 5.83; N, 8.08.

The synthetic schemes of **1680**, **1908**, **1910**, **1714**, **1619**, **1622**, **1737**, **1895**, **1463**, **1670**, and **1713** are published in Moianos, et al (5).

**References**

1. **Michigami K, Murakami H, Nakamura T, Hayama N, Takemoto Y.** 2019. Catalytic asymmetric aza-Michael addition of fumaric monoacids with multifunctional thiourea/boronic acids. Org Biomol Chem **17:**2331-2335.

2. **Longyang Dian SW, Daisy Zhang-Negrerie, Yunfei Du.** 2015. Organocatalytic Radical Involved Oxidative Cross-Coupling of N-Hydroxyphthalimide with Benzylic and Allylic Hydrocarbons. Advanced Synthesis and Catalysis **357:**3836-3842.

3. **Wang X, Lu N, Yang Q, Gong D, Lin C, Zhang S, Xi M, Gao Y, Wei L, Guo Q, You Q.** 2011. Studies on chemical modification and biology of a natural product, gambogic acid (III): determination of the essential pharmacophore for biological activity. Eur J Med Chem **46:**1280-1290.

4. **Grottelli S, Annunziato G, Pampalone G, Pieroni M, Dindo M, Ferlenghi F, Costantino G, Cellini B.** 2022. Identification of Human Alanine-Glyoxylate Aminotransferase Ligands as Pharmacological Chaperones for Variants Associated with Primary Hyperoxaluria Type 1. J Med Chem **65:**9718-9734.

5. **Moianos D, Makri M, Prifti GM, Chiotellis A, Pappas A, Woodson ME, Tajwar R, Tavis JE, Zoidis G.** 2024. N-Hydroxypiridinedione: A Privileged Heterocycle for Targeting the HBV RNase H. Molecules **29**:2942.
